# Dynamic gene regulatory network inference from single-cell data using optimal transport

**DOI:** 10.1093/bioinformatics/btaf394

**Published:** 2025-07-12

**Authors:** François Lamoline, Isabel Haasler, Johan Karlsson, Jorge Gonçalves, Atte Aalto

**Affiliations:** Luxembourg Centre for Systems Biomedicine, University of Luxembourg, Belvaux, L-4367, Luxembourg; Department of Information Technology, Uppsala University, Uppsala, 751 05, Sweden; Department of Mathematics, KTH Royal Institute of Technology, Stockholm, 100 44, Sweden; Department of Mathematics, KTH Royal Institute of Technology, Stockholm, 100 44, Sweden; Luxembourg Centre for Systems Biomedicine, University of Luxembourg, Belvaux, L-4367, Luxembourg; Department of Plant Sciences, University of Cambridge, Cambridge, CB2 3EA, United Kingdom; Luxembourg Centre for Systems Biomedicine, University of Luxembourg, Belvaux, L-4367, Luxembourg; Department of Cancer Research, Luxembourg Institute of Health, Strassen, L-1445, Luxembourg

## Abstract

**Motivation:**

Modelling gene expression is a central problem in systems biology. Single-cell technologies have revolutionized the field by enabling sequencing at the resolution of individual cells. This results in a much richer data compared to what is obtained by bulk technologies, offering new possibilities and challenges for gene regulatory network inference.

**Results:**

In this work, we introduce GRIT (gene regulation inference by transport)—a method to fit a differential equation model and to infer gene regulatory networks from single-cell data using the theory of optimal transport. The idea consists in tracking the evolution of the cell distribution over time and finding the system whose temporal marginals minimize the transport cost with the observations. GRIT is finally used to identify genes and pathways affected by two Parkinson’s disease associated mutations.

**Availability and implementation:**

Matlab implementation of the method and code for data generation are at gitlab.com/uniluxembourg/lcsb/systems-control/grit together with a user guide. A snapshot of the code used for the results of this article is at doi: 10.5281/zenodo.15582432.

## 1 Introduction

Exploration of the regulatory relationships between genes is a central problem in systems biology and medicine. Understanding the regulation of cellular functions is key to the discovery of the molecular mechanisms behind diseases and treatments (Boyle *et al.* 2017). Single-cell techniques now allow sequencing at the resolution of individual cells for a large number of cells at a time. However, the measurements are destructive, which prevents observing a cell over time. Instead, the data consist of population snapshots at different times.

Inference of gene regulatory networks (GRNs) from bulk time series data has been a longstanding problem in systems biology (Huynh-Thu *et al.* 2010, Marbach *et al.* 2010, Aalto *et al.* 2020). Inference of dynamical models from time series data is also a common problem in many fields of engineering. However, compared to most engineering applications, in molecular biology, data collection is expensive and laborious. Therefore, the main challenge in GRN inference from bulk time series data is to deal with the small amount of data, in particular, given the high dimension of the problem. In the highly simplified framework of discrete-time linear systems with full state measurements, it is well known that to infer an *n*-dimensional system, at least n+1 time points are needed. This requirement is never satisfied with bulk transcriptomics data, unless the set of included genes is heavily reduced. A common workaround is to impose sparsity constraints to the inferred models.

Time course single-cell data, in contrast, reveal the time evolution of the full distribution of the cell population. Aalto *et al.* (2022) show that three time points of such population snapshot data are sufficient for unique identifiability of a discrete-time linear system, regardless of the system dimension. Despite the overly simple model class, this result gives hope that single-cell data can accelerate transcriptomics research. Methods for GRN inference from single-cell data have been proposed based on, e.g. information-theoretic considerations [PIDC by Chan *et al.* (2017), scNME by Li *et al.* (2023)], regression [GENIE3 by Huynh-Thu *et al.* (2010), GRNBoost by Moerman *et al.* (2019)], co-expression (Moignard *et al.* 2013), and dynamical models [SCODE by Matsumoto *et al.* (2017), GRISLI by Aubin-Frankowski and Vert (2020), Cardamom by Ventre *et al.* (2023), and others (Ocone *et al.* 2015, Aalto and Gonçalves 2019, Fang *et al.* 2024)]. Information-theoretic, and regression-based methods do not use temporal information, whereas dynamical model-based methods require temporal information, either in the form of pseudotime [SCODE, GRISLI (Ocone *et al.* 2015, Aalto and Gonçalves 2019)] or actual measurement times or at least their temporal ordering [Cardamom, GRISLI (Aalto and Gonçalves 2019, Fang *et al.* 2024)]. In a benchmarking study by Pratapa *et al.* (2020a), regression-based methods seemed to be the most consistent, and were the best performers in the inference task from real scRNA-Seq data. Regression-based methods—even though demonstrated to have good performance—are heuristic, and not rooted in physical considerations. Of the methods for inferring dynamical systems from single-cell data, SCODE by Matsumoto *et al.* (2017) and the method presented by Ocone *et al.* (2015) are based on transforming the data into a pseudotime series. SCODE then fits to pseudotime data continuous trajectories represented as combinations of the system’s dynamical modes. In the work of Ocone *et al.* (2015), dynamics governed by the Hill equation are fitted to pseudotime data. GRISLI by Aubin-Frankowski and Vert (2020) is based on estimating a velocity for each cell using a kernel approach, and solving a regression problem with these velocities. Fang *et al.* (2024) fit a chemical master equation to observed distributions, but this method is not developed for snapshot data. Cardamom by Ventre *et al.* (2023) develops a mechanistic model of gene expression that leads to a specific form of the expression distribution that is then fitted to data. In this work, we propose to use optimal transport (OT) cost to evaluate the model’s fit to observed data.

OT provides a natural way to compare distributions. While the original formulation of the OT problem by Monge (1781) and Kantorovich (1942) discussed a very concrete problem of moving earth from an initial configuration to a target configuration, more recently OT has found numerous applications ranging from machine learning (Kolouri *et al.* 2017) to imaging, probability theory (Rigollet and Weed 2018) as well as systems theory (Chen *et al.* 2016a, 2016b, Chen and Karlsson 2018, Haasler *et al.* 2021). With the advent of single-cell data, OT has also found several applications in systems biology. Hashimoto *et al.* (2016), Bunne *et al.* (2022), Yachimura *et al.* (2024), and Zhang *et al.* (2021) use OT to infer cell dynamics driven by a gradient flow governed by a potential function (analogous to the Waddington landscape). Schiebinger *et al.* (2019) use OT to find couplings between consecutive time points to identify single-cell trajectories in the form of ancestor and descendant distributions. Huizing *et al.* (2022) develop a metric for cell–cell similarity by interpreting each cell’s feature profile as a probability distribution, and defining a cost of transport between features. Huizing *et al.* (2023) use OT on paired single-cell multi-omics datasets as a loss function in a matrix factorization approach. Bunne *et al.* (2023) couple a perturbed cell group using OT with a control group to predict perturbation responses. Cang and Nie (2020) and Cang *et al.* (2023) use OT to align single-cell data with spatial transcriptomics data, and further, to match ligand and receptor distributions to study cell–cell communication.

This work introduces GRIT (gene regulation inference by transport theory), a method based on fitting a linear differential equation model to the observed data using the concept of OT. The method works by propagating cells measured at a certain time $T_{k}$ through a candidate model, and calculating the transport cost between the propagated population and the cell population measured at the next time point $T_{k+1}$. The goal is to determine the model that minimizes the OT cost. Unlike regression-based models, e.g. differential equation models are inherently causal. GRIT also naturally extends to additional challenges related to GRN inference, namely inference of perturbation targets and mutation effects in the network. Moreover, we prove a consistency result for the method, stating that if the data were generated from a linear discrete-time system, then the true system is the unique global minimizer of the defined transport cost. Recent preprints by Shen *et al.* (2024), Zhang (2024), and Guan *et al.* (2024) have emerged that propose very similar approaches as GRIT for inferring differential equation models from single-cell data. That is, they propose iteratively solving an OT problem and a model fitting problem.

GRIT is first validated using synthetic data to study the effect of various details on its performance. GRIT is then applied on the BEELINE benchmarking pipeline proposed by Pratapa *et al.* (2020a) to compare it with state-of-the-art methods. GRIT demonstrates robust and good performance, in particular outperforming state-of-the-art methods when applied to simulated data. Finally, GRIT is applied on two real datasets by Novak *et al.* (2022) and Walter *et al.* (2021), generated with the aim to study the effect of different mutations (*LRRK2*-G2019S and *PINK1*-I368N) associated with Parkinson’s disease on neuron differentiation. GRIT identifies perturbation targets corresponding to the mutations as well as enriched pathways and we compare the findings with existing literature.

## 2 Materials and methods

This section introduces the essential parts of GRIT. A more extensive description, rationale for some modelling choices, and background on OT are in Note S1, available as supplementary data at *Bioinformatics* online.

### 2.1 Gene expression model

Cells are modelled as individuals evolving in the gene expression space. The gene expression levels of *n* genes form the cell’s state vector *x*. It is assumed to be governed by the stochastic linear differential equation


(1) $$dx(t)=(Ax(t)+b)dt+\sqrt{\epsilon }dw(t),\;x(0)∼P_{0}$$

where *A* is a sparse matrix, *b* is a constant load, *w* is a standard Brownian motion, and ε>0 is the noise intensity, assumed to be constant. Biologically, *A* contains the regulatory parameters of the transcription factors (TFs). The model for individual cell dynamics gives rise to a time-varying probability distribution for the cell population, and the single-cell data consist of samples of this distribution at different times (Hasenauer *et al.* 2011).

### 2.2 Optimal transport

The OT theory is a natural tool for finding a coupling between point clouds, and measuring the distance between them. Say $P=[p_{1},\ldots ,p_{m_{P}}]\in R^{n\times m_{P}}$ is a matrix containing the points of one point cloud consisting of $m_{P}$ points and $Q=[q_{1},\ldots ,q_{m_{Q}}]\in R^{n\times m_{Q}}$ another point cloud consisting of $m_{Q}$ points. Using squared distance as the transportation cost, the OT problem (with entropy regularization) is


(2) $$\begin{array}{c} W(P,Q):=\min_{M}\sum_{i=1}^{m_{P}}\sum_{j=1}^{m_{Q}}\left[∥p_{i}-q_{j}{∥}^{2}M_{i,j}+\varepsilon M_{i,j}\;\text{log}(M_{i,j})\right]\\ \text{such}\;\text{that}\;M1=\mu_{P}\;\text{and}\;M^{⊤}1=\mu_{Q}. \end{array}$$

where $\mu_{P}\in R^{m_{P}}$ and $\mu_{Q}\in R^{m_{Q}}$ are the weights of the samples in the respective distributions. The transport plan $M\in R_{+}^{m_{P}\times m_{Q}}$ gives a coupling between the points in the two sets that minimizes the total cost of transportation. The minimal value is used as a measure of the quality of fit between *P* and *Q*. The entropy regularization is fairly standard in OT problems with several functions (Peyré and Cuturi 2019, Chapter 4). For example, it enables efficient numerical solution by the so-called Sinkhorn iterations (Cuturi 2013), but in our approach it also accounts for noise in the cell dynamics. It is not an accident that we use the same symbol ε for both noise intensity in (1) and regularization strength in (2).

### 2.3 Model identification

**Figure 1.**
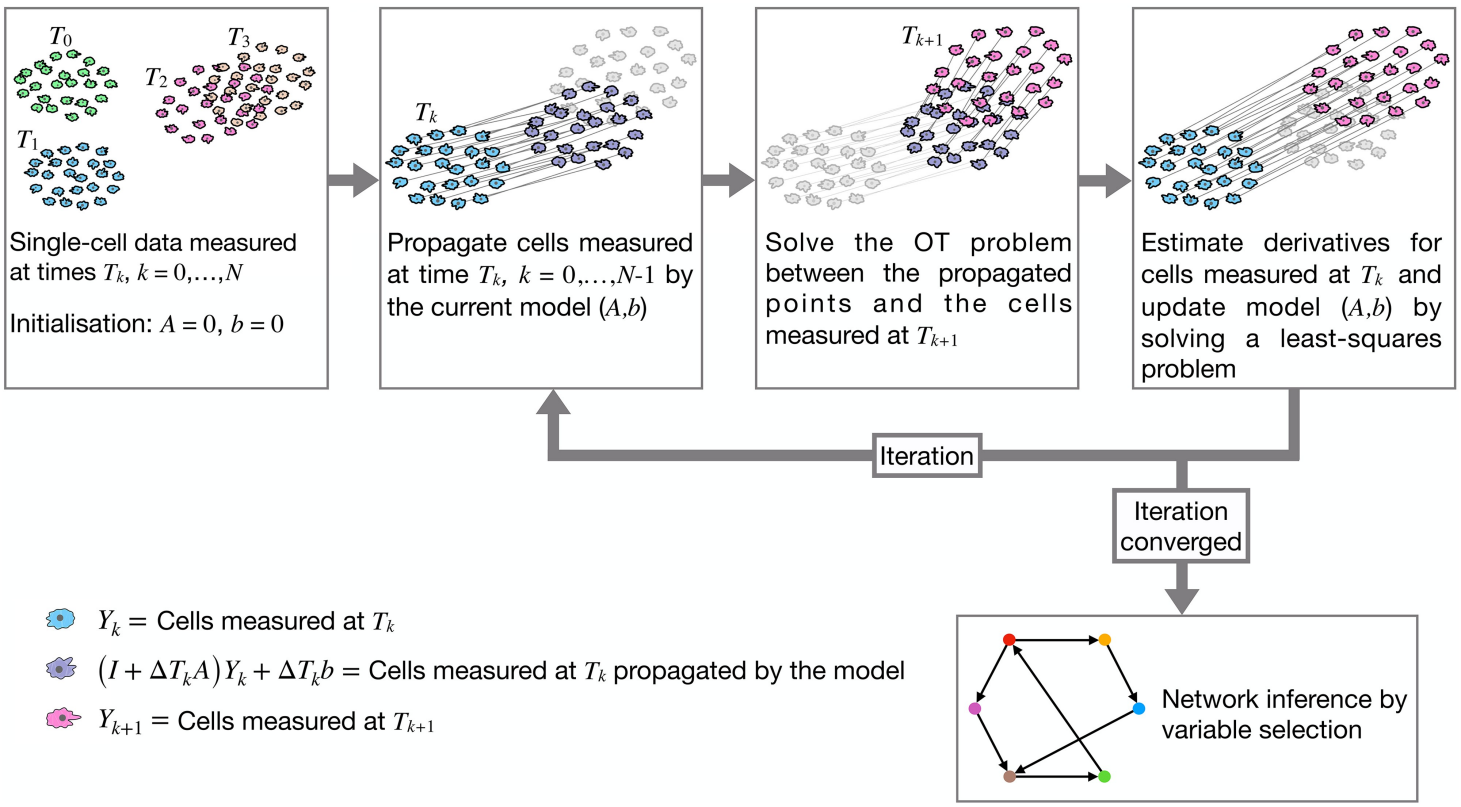
Method pipeline: The model identification iteration is run until convergence. The iteration can be interpreted as a block coordinate descent for solving (3), alternating between minimization with respect to the transport plans $M_{k}$ and the model (A,b). The results are then used in a separate variable selection step to obtain confidence scores for GRN link existence.

As summarized in Fig. 1, the cell distribution measured at time $T_{k-1}$ is first propagated through system (1). Then, this propagated distribution is compared with the population measured at $T_{k}$ by calculating the OT cost between the populations. To obtain a mathematically tractable problem, the propagation is done using a first-order (Euler) discretization of (1). If $Y_{k-1}$ is the expression matrix for the population measured at time $T_{k-1}$, the propagated matrix is given by $(I+\Delta T_{k}A)Y_{k-1}+\Delta T_{k}b$. The cost function for evaluating a model (A,b) is obtained by combining all time points:


(3) $$\begin{array}{c} \underset{A,b}{\min}J(A,b)=\underset{A,b}{\min}\sum_{k=1}^{N}\frac{1}{\Delta T_{k}}W((I+\Delta T_{k}A)Y_{k-1}+\Delta T_{k}b,Y_{k})\\ +∥A\Lambda_{A}^{1/2}{∥}_{F}^{2}+\lambda_{b}∥b{∥}^{2}. \end{array}$$

The diagonal matrix $\Lambda_{A}$ and $\lambda_{b}$ are regularization parameters. The weight $1/\Delta T_{k}$ in the sum is related to the effect of noise *dw* in (1). Note that the calculation of the transport cost *W* involves the solution of a minimization problem (2). The combined optimization problem for the system variables (A,b), and the transport plans $M_{k}$ for k=1,…,N (see Eq. (8) in Note S1, available as supplementary data at *Bioinformatics* online) is solved by a block coordinate-descent type algorithm, illustrated in Fig. 1. When *A* and *b* are fixed, the minimization problem is a standard OT problem that is solved by Sinkhorn iterations. When, in turn, the transport plans are fixed, the minimization problem is quadratic with respect to (A,b). The combined problem is solved by alternately minimizing with respect to the two variables.

Our main objective is to use the OT cost as a goodness-of-fit measure for the dynamical system *A*, *b*. However, the transport plans $M_{k}$ obtained for each time transition $T_{k-1}\to T_{k}$ are interesting on their own. They can be used to identify ancestor and descendant cells as in the Waddington-OT work by Schiebinger *et al.* (2019). The transport plan $M_{k}$ can also be used to define a target point at time $T_{k}$ for each cell measured at time $T_{k-1}$. With matrix notation, the target points for all cells in the matrix $Y_{k-1}$ are given by $Y_{k}M_{k}^{⊤}$. These target points can then be used to estimate derivatives for the cells by a difference quotient $(Y_{k}M_{k}^{⊤}-Y_{k-1})/\Delta T_{k}$, corresponding to RNA-velocity (La Manno *et al.* 2018) (at least when $\Delta T_{k}$ is not too large).

### 2.4 GRN inference

The dynamical system parameterized by the matrix *A* and the vector *b* already describe the regulatory structures between genes, and could be used for simulation studies or observability analysis following Hasnain *et al.* (2023). In principle, the higher the absolute magnitude of an element in the *A* matrix, the stronger is the corresponding regulation. However, the matrix element magnitudes are sensitive to gene expression level scales, and they are not necessarily the best measure of the confidence on the existence of a regulatory effect. Therefore, once the model identification algorithm has converged, the results are used in a separate variable selection step, whose results are interpreted as confidence scores for the existence of a regulatory link. The additional variable selection step is based on a greedy forward-backward sweep [inspired by the work of Zhang (2011) to which we also refer for details on the greedy approach] applied on the regression problem obtained from (3) by keeping the transport plans $M_{k}$ fixed. To initialize the backward greedy algorithm, the initial set of regressors is selected based on a combination of gene–gene correlations and a forward greedy algorithm, i.e. at every step adding to the active regressor set the regressor yielding the highest decrease in the cost function. The backward greedy algorithm is then carried out, i.e. at every step removing the regressor whose removal yields the smallest increase to the cost function. The confidence scores for the existence of links are based on cost function increments during the backward sweep.

### 2.5 Further details on the method and its use

External perturbations can be introduced by adding a second vector *b* to (1), i.e. only active when the perturbation is active. Targets of this perturbation can then be inferred as part of the network inference procedure. Perturbation target inference is described in more detail in Note S1.5, available as supplementary data at *Bioinformatics* online. Effects of a mutation in a known gene can be inferred as well. In that case, the column of the *A* matrix corresponding to the mutated gene is allowed to differ between the mutation and control datasets. Mutation effect inference is described in more detail in Note S1.6, available as supplementary data at *Bioinformatics* online.

In some single-cell datasets, the population splits into separate subgroups over time, e.g. differentiating into different cell types. The user is advised to identify first whether there is branching. In such cases, branching dynamics should be identified first, and branch labels are given as an input to GRIT. The handling of branching dynamics is described in Note S1.7, available as supplementary data at *Bioinformatics* online. Branch labels can be obtained using a suitable pseudotime inference method, like Slingshot by Street *et al.* (2018), or alternatively, using the GRIT branch labelling scheme described in Note S1.8, available as supplementary data at *Bioinformatics* online. In case the branches are related to subpopulations that evolve separately during the entire experiment timeline (e.g. different cell types), it may be beneficial to identify the population of interest, and restrict analysis on the corresponding branch while filtering out all other cells.

GRIT can also output signed predictions where the sign indicates whether the regulation is an activation or inhibition. Signs are based on the signs of the inferred *A* matrix.

When using GRIT, it is advised to reduce the number of genes by filtering out genes whose variability over time is small. We leave it to the user to decide on the method and thresholds, but following the pipeline we use in the analysis of the LRRK2 and PINK1 datasets is one feasible strategy. Cell cycle removal can be done, but if the cell cycle is in steady state across the dataset, the cell cycle genes will likely be filtered out anyway.

The data should consist of several (preferably at least three) snapshots at different times. In case dynamics are captured in a single snapshot (like in BEELINE mHSC datasets), pseudotime can be used to generate snapshot data. We recommend to make sure that the generated snapshots have at least 120 cells (and preferably more) to ensure that the snapshots span the reduced-dimension space used in the OT problem. However, we advise to use the actual snapshots if available.

### 2.6 Datasets and method validation

We first validate GRIT on synthetic data generated from a 10D discrete-time linear system corresponding to the method design. Then, complexity is increased stepwise moving to continuous-time systems, nonlinear systems (using Michaelis–Menten kinetics), and finally to a model with hidden states corresponding to protein dynamics. Details of data generation are in Note S5, available as supplementary data at *Bioinformatics* online.

Second validation is done using the BEELINE benchmarking pipeline by Pratapa *et al.* (2020a, 2020b), consisting of three types of datasets. The “synthetic” dataset consists of simulated data from six systems that are purpose-designed to reproduce certain qualitative behaviors, such as bifurcation, periodic dynamics, etc. The “curated” dataset consists of simulated data from four models from literature, created to mimic certain biological processes. Finally, five real single-cell RNA-Seq datasets are included.

After validation, we analyse experimental datasets on which our biological objective is to look into genetic mechanisms of Parkinson’s disease (PD). In particular, we look into two datasets studying the effect of PD-associated mutations on the neuron development, namely the *PINK1*-I368N mutation (Novak *et al.* 2022) and the *LRRK2*-G2019S mutation (Walter *et al.* 2021) (referred to as the *PINK1* and *LRRK2* datasets in the article). These datasets contain single-cell RNA-seq data for stem cells differentiating into dopaminergic neurons. For details on the BEELINE and the *LRRK2* and *PINK1* datasets, see Note S3.1, available as supplementary data at *Bioinformatics* online.

For performance evaluation, standard scores are used, namely the area under the receiver operating characteristics curve (AUROC), and the area under the precision–recall curve (AUPR). The AUPR emphasizes high-confidence predictions and it should be regarded as the primary metric due to network sparsity. In the context of the BEELINE benchmark, we use the performance metrics proposed in BEELINE. In the main text, we show only summary results based on method ranking, comparing with other methods benchmarked in BEELINE. See Note S3.2, available as supplementary data at *Bioinformatics* online, for details.

## 3 Results

### 3.1 Theoretical consistency result

The use of a first-order discretization in (3) effectively renders the method a discrete-time system identification scheme. If, indeed, the data originate from a discrete-time system, then the following result holds.

Box 1. Consistency result

Assume that the data are produced by a discrete-time system

$$\begin{array}{c} x_{k}=(I+\Delta T\bar{A})x_{k-1}+\Delta T\bar{b}+\sqrt{\Delta T\varepsilon }w_{k},\\ x_{0}∼N(m_{0},P_{0}) \end{array}$$

and data on at least three time points kΔT with k=0,1,…,N is measured. With the number of cells measured on each time point tending to infinity, the true system $\left(\bar{A},\bar{b}\right)$ is the unique global minimizer of the cost function (3).

The precise statement of this result and its proof are in Note S4, available as supplementary data at *Bioinformatics* online. The proof relies on recent results on entropy-regularized OT by Janati *et al.* (2020) and Mena and Niles-Weed (2019) and on an identifiability result for uniqueness by Aalto *et al.* (2022). Interestingly, as already mentioned, the entropy regularization acts as a noise deconvolution for the stochastic dynamics. This idea of entropy regularization as noise deconvolution holds more generally and it has been explored by Rigollet and Weed (2018).

### 3.2 Performance on linear discrete-time dynamics

**Figure 2.**
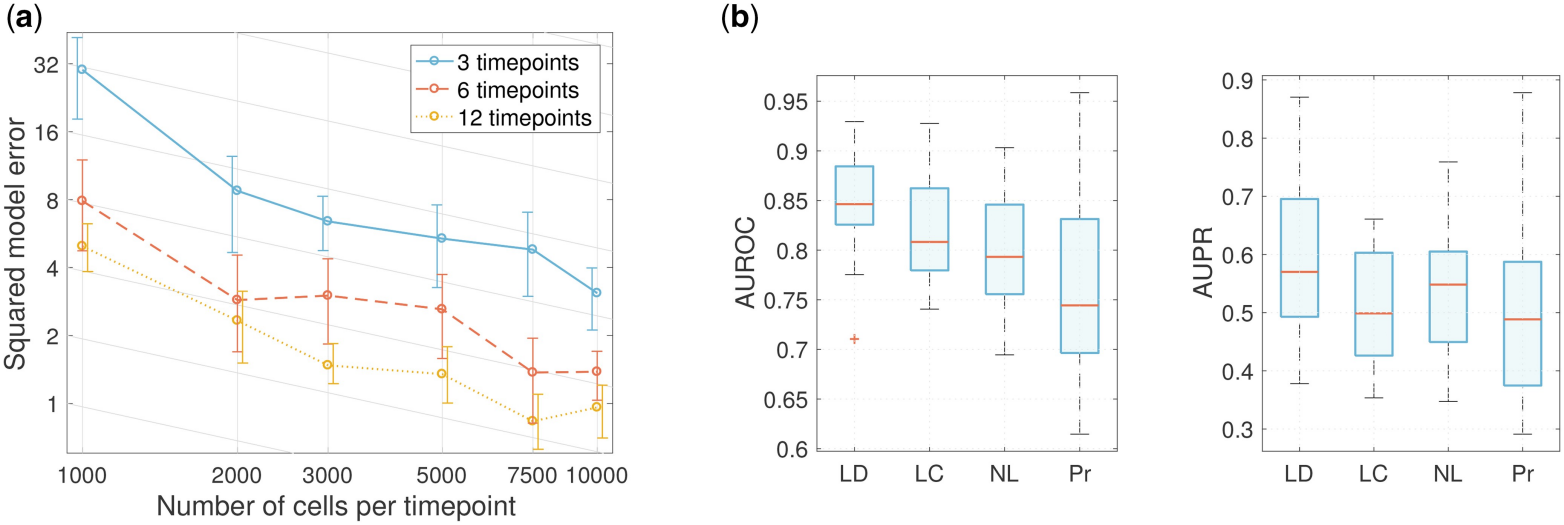
(a) The squared model error $∥[A,b]-[\bar{A},\bar{b}]{∥}_{F}^{2}$ with data simulated from a linear discrete-time system with varying number of timepoints and number of cells per timepoint. The plot is in logarithmic scale and it shows the mean and 80th percentiles obtained from five replicates. The sloped gridlines correspond to a decay $m^{-1/2}$. (b) The AUROC and AUPR scores from 20 replicates with data simulated from linear discrete-time system (LD), linear continuous-time system (LC), nonlinear system (NL), or system with non-observed protein concentrations (Pr).

To investigate GRIT’s performance in the framework of the consistency result, it is here applied on data generated from a 10D linear discrete-time system. The consistency result holds when the number of cells measured at each time point tends to infinity. However, it should be noted that the result only guarantees that the true system is the unique global minimizer of the cost function (3), but it does not guarantee convergence. To study convergence properties, GRIT was applied to data with either three, six, or 12 time points, with 1000–10000 cells per time point. In this experiment, the method was tuned to correspond precisely to the assumptions of the theorem, i.e. we set $\Lambda_{A}=\lambda_{b}=0$, and the entropy regularization parameter ε was set to the noise intensity used in the data generation. The results are shown in Fig. 2a in terms of squared Frobenius norm between the estimated and true (A,b). The estimated system appears to converge towards the true system such that $∥[A,b]-[\bar{A},\bar{b}]{∥}_{F}^{2}\propto m^{-1/2}$, where *m* is the number of cells per timepoint. Incidentally, this is precisely the convergence rate (in matrix norm) of the empirical covariance matrix (Vershynin 2012). The computation time is polynomially increasing ($\propto m^{2.42}$) with the number of cells (Fig. S13, available as supplementary data at *Bioinformatics* online).

### 3.3 Effect of continuous time, nonlinear dynamics, and hidden states

The framework of linear discrete-time dynamics is a crude simplification of underlying biological processes. Now we go step-by-step towards more realistic data generation setup and study how GRIT’s performance changes. First, continuous-time simulations are done instead of discrete-time. Then, the linear dynamics are replaced by nonlinear Michaelis–Menten kinetics including transcription saturation and more realistic inhibition action. Moreover, the constant-intensity white noise used with linear systems is replaced by the more realistic state-dependent Langevin noise (Gillespie 2000). Finally, hidden states mimicking protein concentrations are introduced, one protein corresponding to each gene with linear dynamics for protein translation and degradation (Note S5, available as supplementary data at *Bioinformatics* online).

Results on 20 replicates are shown in Fig. 2b. In this experiment, six time points were simulated with equally spaced measurement times. There is a clear drop in performance when moving from discrete- to continuous-time systems. This is somewhat expected when moving away from the precise model class for which the method was developed. Interestingly, there is no significant decrease when moving from linear to nonlinear dynamics. Performance drops again when hidden states are introduced. Moreover, the performance variability is quite high, most likely due to the randomization of the parameters in the protein dynamics. Slower protein dynamics cause delays that might blur the observability of regulatory interactions.

This experiment was also used to test the effect of using single-cell data instead of corresponding bulk data. To this end, GRIT was applied on data where the expression vector of each cell was replaced by the average expression level of the corresponding time point. This corresponds to running GRIT’s network inference step on bulk data. The performance is far worse than with single-cell data (Fig. S4a, available as supplementary data at *Bioinformatics* online). In addition, the variable selection step for network inference was validated. Using the absolute values of the *A* matrix entries as the output, the results are reasonable but still inferior to GRIT (Fig. S4b, available as supplementary data at *Bioinformatics* online).

### 3.4 Validation by BEELINE pipeline

**Figure 3.**
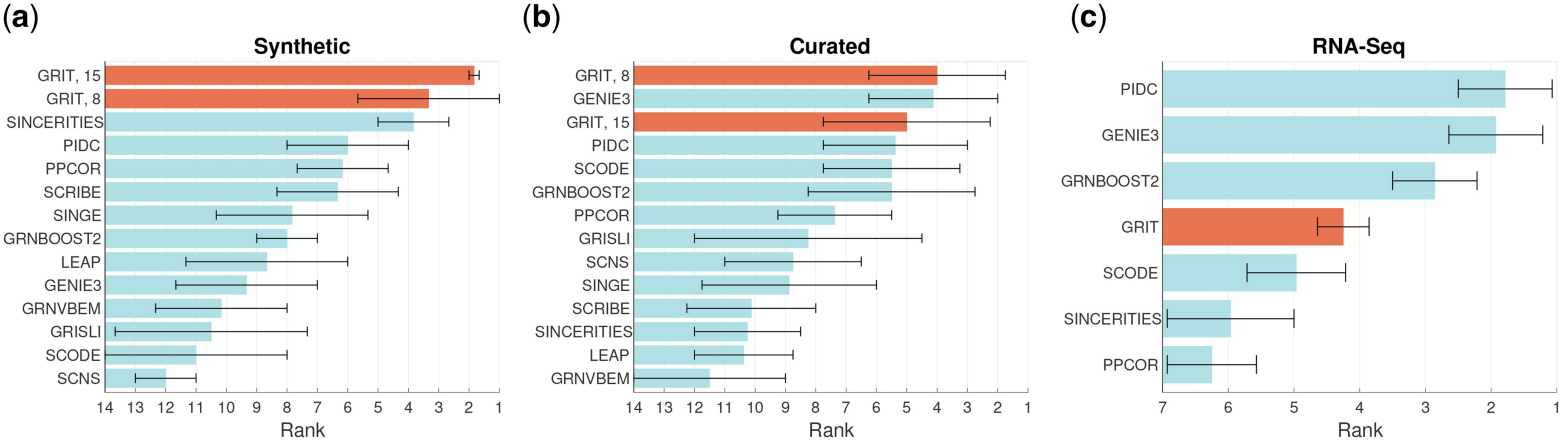
Results summary on the BEELINE benchmarking pipeline for (a) the synthetic dataset, (b) curated dataset, and (c) the RNA-Seq data. The bars show the average rank of each method in the different tasks, and the error bars indicate the means of the bottom 50th percentile and the top 50th percentile.

BEELINE was proposed by Pratapa *et al.* (2020a) as a systematic pipeline to evaluate GRN inference methods from single-cell data. It consists of both simulated datasets with known ground truth networks, and real data with ground truth networks either extracted from STRING database or obtained by ChIP-Seq. Summary results of GRIT on the BEELINE pipeline are shown in Fig. 3 (full results are in Figs S5–S12 and Table S1, available as supplementary data at *Bioinformatics* online).

The simulated data in BEELINE are not given in snapshots at different time points, but we have generated time points by ordering the cells by pseudotime and then dividing the data into 8 or 15 timepoints. These correspond to the results labelled “GRIT, 8” and “GRIT, 15” in Fig. 3a and b. Overall, this number of time points did not change much the results (see Figs S5 and S6, available as supplementary data at *Bioinformatics* online), with the exception of the LL system in the synthetic dataset. This system is a cascade of 18 genes that are activated one after the other in very rapid succession. Eight timepoints are not sufficient to properly capture these fast dynamics. A closer inspection of the results revealed that GRIT inferred many indirect regulations, giving high confidence to a regulation A→C when the true regulation chain was A→B→C. In three of the six systems (BF, BFC, and TF), GRIT clearly outperforms other methods. Interestingly, these are the systems with branching dynamics, which GRIT can clearly handle well despite its reliance on linear dynamics. However, branch information should be given to the method to improve its performance (which was also done in the BEELINE evaluation). With the LI and CY systems, GRIT is among the top performers attaining nearly perfect score.

In the four inference tasks with the curated datasets, GRIT is the top performer in one task (GSD), among top performers in one (HSC), and slightly behind the top performers in two tasks (mCAD and VSC). In the mCAD task, very few methods get a score better than random guessing (AUPR-ratio >1). In the VSC task, data quickly converge to the statistical steady state with dynamics only in the very beginning. This may favour information theory- and regression-based methods. Re-defining the simulation time scale might improve performance for all methods, but particularly for those based on dynamical modelling like GRIT.

Regression-based methods are the best performers in the tasks using real scRNA-Seq data. Curated network databases, like STRING, may have a bias towards correlation- and regression-based methods, since earlier discoveries are often based on co-expression studies. Moreover, STRING networks are based on protein interactions, and are therefore symmetric (except that links from non-TF genes have been deleted), thereby favouring regression-based methods. Regression-based methods are also possibly more robust against data issues due to limitations of current sequencing technologies. GRIT’s good performance with the cleaner simulated data gives hope of performance improvement as sequencing technology advances. Sensitivity analysis on the entropy regularization parameter ε in (2) and on the regression regularization parameters $\Lambda_{A}$ and $\lambda_{b}$ in (3) reveals that their impact is rather small on the results of the BEELINE RNA-seq data (Fig. S11, available as supplementary data at *Bioinformatics* online) as is the impact following different transformations (Fig. S12, available as supplementary data at *Bioinformatics* online). The results shown in Fig. 3c are obtained with log-transformed data [as provided by Pratapa *et al.* (2020b)].

Following Pratapa *et al.* (2020a), the results on the synthetic data include the cases with 2000 or 5000 cells, and the results on the curated data include only cases without dropouts. The results with smaller number of cells and with dropouts are in Fig. S9, available as supplementary data at *Bioinformatics* online. The synthetic dataset was used to evaluate signed predictions, for which results are in Fig. S10, available as supplementary data at *Bioinformatics* online. The signs are accurately captured by GRIT. Computation times with varying dimension and number of cells are in Fig. S13, available as supplementary data at *Bioinformatics* online, and a comparison with other methods is in Table S4, available as supplementary data at *Bioinformatics* online.

### 3.5 Perturbation target identification for *PINK1* and *LRRK2* mutations

**Figure 4.**
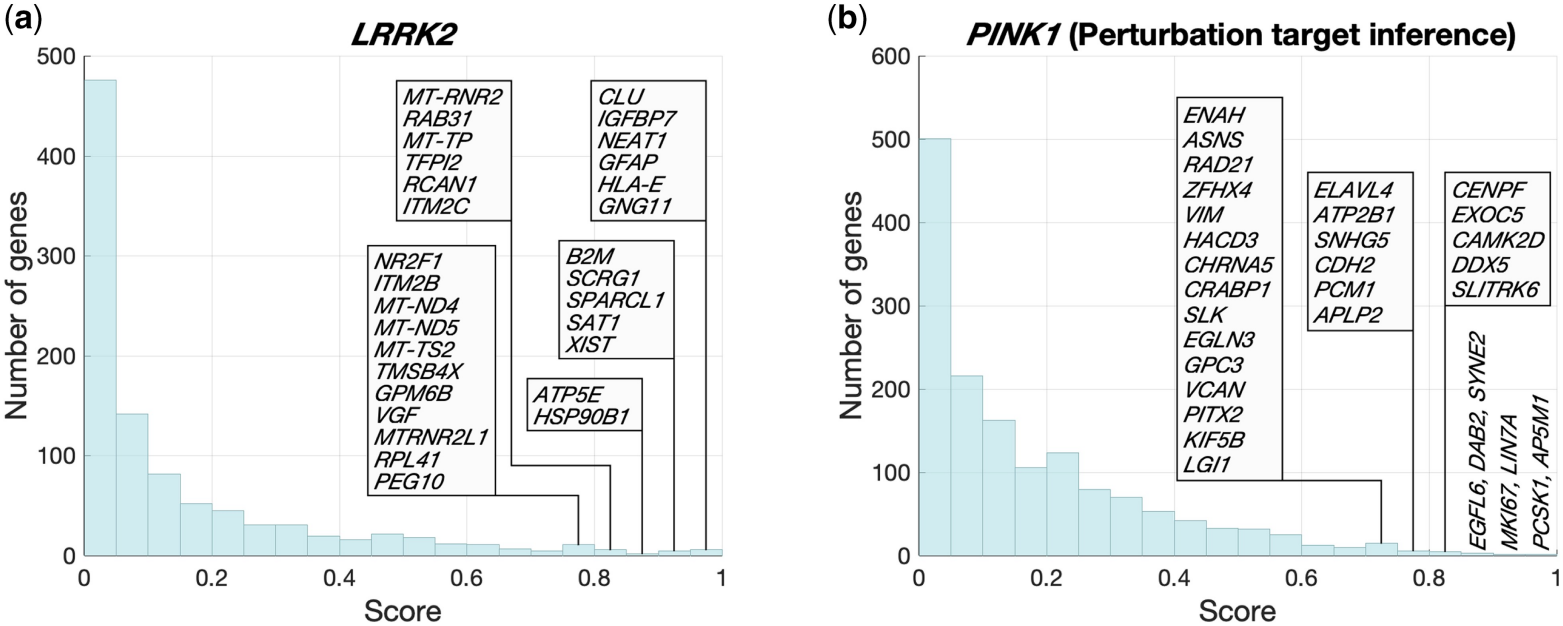
Histogram of perturbation target scores with high-confidence genes indicated for the *LRRK2* mutation (a) and for the *PINK1* mutation (b).

In the analysis of the *PINK1* and *LRRK2* results, the focus is put on perturbation target inference, i.e. discovery of genes whose dynamics have been directly affected by the mutations. Since *LRRK2* itself is expressed in only very few cells in the data, this experiment had to be treated as a perturbation dataset. *PINK1* is better expressed, but it is not among the 2000 most highly varying genes. *PINK1* is nevertheless included in the analysis. GRIT is applied to the *PINK1* dataset using both the perturbation target inference and mutation effect inference approaches. The results for the mutation effect inference are shown and discussed in Note S2.4, available as supplementary data at *Bioinformatics* online. Figure 4 shows the histograms of gene scores for being affected by the perturbation and lists the most highly scoring genes.

The top target candidate in the *LRRK2* case is *CLU*, which has been identified as a marker for a cell type heavily affected by PD, along with *PEG10* (Martirosyan *et al.* 2024). The role of *NEAT1* in PD development has attracted interest (Boros *et al.* 2021), although it has been suggested that it acts as a *LRRK2* inhibitor. *GNG11* was suggested by Moradi *et al.* (2022) as a PD biomarker in a network-based study on PD patients carrying the *LRRK2*-G2019S mutation. *SAT1* is PD-associated (Lewandowski *et al.* 2010), although without a known direct connection with *LRRK2*. *XIST* has been reported by Zhou *et al.* (2021) to modulate the *LRRK2* signalling pathway and to accelerate PD development. *HSP90B1* is a chaperone protein that interacts with *LRRK2* (Zagare *et al.* 2022). *LRRK2* phosphorylates *RCAN1*, but there is no evidence on transcriptional regulation (Han *et al.* 2017). *NR2F1* is the main gene identified by Walter *et al.* (2021) (where the *LRRK2* dataset originates from) in the mechanism how *LRRK2* influences dopaminergic differentiation. *VGF* secretion is regulated by *LRRK2* (Filippini *et al.* 2023).

Among the top perturbation target candidates in the *PINK1* case with perturbation target inference is *MKI67*, which is a proliferation-related gene inhibited by *PINK1* (Jia *et al.* 2021, Fig. 4). *ELAVL4* is PD-associated, in particular, it is related to the age of PD onset (DeStefano *et al.* 2008). Interactions between *PINK1* and *DDX5*, *PCM1*, *ASNS*, and *VIM* are reported by Guardia-Laguarta *et al.* (2019, Fig. 1–1). Some of the identified genes interact with other well-known PD-associated genes, namely *SNHG5* interacts with *LRRK2* (Novak *et al.* 2022) and *GPC3* with *DJ-1* (*PARK7*) (Novak *et al.* 2022).

To gain overarching insight into the results, pathway enrichment analysis was performed with correction for bias due to the selection of genes in the analysis and accounting for the scores (see Note S3.3, available as supplementary data at *Bioinformatics* online). The results of the analysis for the perturbation target inference results are in Table S2, available as supplementary data at *Bioinformatics* online, for the *LRRK2* case and Table S3, available as supplementary data at *Bioinformatics* online, for the *PINK1* case.

In the *LRRK2* results, all of the three statistically significantly enriched pathways have a high overlap with each other. There is a set of 32 genes that belong to all three terms. Out of these genes, 24 are in top-400 of GRIT’s results. To zoom in on these genes from the fairly high level KEGG pathways, these genes were fed to g:profiler with GO pathways (Ashburner *et al.* 2000, Aleksander *et al.* 2023). “Oxidative phosphorylation” is at the top of the results with GO:BP pathways (containing all of the 24 genes with term size 145). Other top results contain “inner mitochondrial membrane protein complex” (GO:CC) (24/162), “respiratory chain complex” (GO:CC) (22/93), and “mitochondrial respirasome” (22/96). The *LRRK2*-G2019S mutation has been shown to impair mitochondrial respiration (Walter *et al.* 2019).

In the enrichment results for the *PINK1* case, “adrenergic signalling in cardiomyocytes” is at the top of the list, followed by “vascular smooth muscle contraction”, “motor proteins”, “melanogenesis”, and “dopaminergic synapse”. While there is some overlap between the significantly enriched terms, there does not appear to be clear clustering, or finer level driving terms. There is a set of 12 genes that belong to more than two of the 14 enriched terms, and nine of these genes are in top-400 of GRIT’s results. Feeding these genes to g:profiler with GO pathways yields “adenylate cyclase activator activity” (GO:MF) as the term with lowest *P*-value, but that contains only three of the nine genes with term size four. The lowest *P*-value for a term containing more genes is obtained for “intracellular calcium ion homeostasis” containing five of the nine genes with term size 310.

The pathway terms appearing in both *LRRK2* and *PINK1* enrichment results are “tuberculosis”, “viral myocarditis”, and “IL-17 signalling pathway”, although mainly not among the statistically significant results after adjustment for multiple hypothesis testing. In particular, IL-17 signalling and viral myocarditis are still interesting finds. The connection of IL-17 (and inflammation in general) and PD has been investigated recently by Storelli *et al.* (2019) and Williams *et al.* (2022). Viral myocarditis is also an inflammatory disease. The connection of *PINK1* and viral myocarditis has been reported earlier by Jin *et al.* (2023), and in general, mitochondrial dysfunction plays an important role in both.

## 4 Discussion

This work proposed GRIT—a GRN inference method based on OT theory. The approach is based on simultaneous inference of the gene expression model and the coupling matrices between cell populations measured at different times. The OT theory is a well-established mathematical framework for comparing particle distributions and it offers a clear interpretation of the optimization problem in terms of finding most likely particle trajectories from ensemble snapshot observations. Moreover, the theoretical tractability of the OT framework allowed us to give a mathematical proof of the method’s consistency—albeit in a very simplified model class of discrete-time linear systems. In addition, GRIT can identify targets of perturbations and mutations, which is a task beyond the scope of correlation and information theory-based methods.

The obtained transport matrices can be used for lineage tracing, i.e. identification of potential ancestor and descendant cells, as in the Waddington-OT approach by Schiebinger *et al.* (2019). In our approach, the model and transport matrices are inferred simultaneously, and the solution of the OT problem is informed by the inferred model, whereas Waddington-OT is based on solving the OT problem directly between cells measured at different times.

GRIT was validated on synthetic and real scRNA-seq datasets, in particular using the BEELINE benchmarking pipeline. On synthetic data, GRIT outperforms state-of-the-art methods. With real single-cell RNA-seq data of the BEELINE benchmark, information-theoretic methods seem to perform best. Several factors may contribute to this result. Information-theoretic methods concatenate all data together ignoring the temporal evolution, and then typically try to solve the expression level of a gene as a function of all other genes. While it is obviously not possible to observe gene dynamics with such an approach, potentially these methods benefit from better robustness against sampling issues and low time resolution which is typical for differentiation experiments, like the ones in the BEELINE benchmark. Methods based on dynamical modelling, like GRIT, could benefit from more frequent sampling.

GRIT was applied on datasets studying the effect of *PINK1* and *LRRK2* mutations on the development of dopaminergic neurons. In particular, the goal was to infer perturbation targets corresponding to the mutations, i.e. to identify genes whose dynamics are directly affected by the mutation. Some of the top genes identified by GRIT are already known to interact with the mutated genes (*PINK1*/*LRRK2*), and many more are known to be differentially expressed between PD patients and healthy individuals. Moreover, results of the pathway enrichment analysis seem highly reasonable. For example, genes related to mitochondrial respiration are enriched in the top predictions in the *LRRK2* case and pathways related to the dopamine system are enriched in the top predictions of the *PINK1* case.

Single-cell techniques are not able to capture all RNAs from cells. Consequently, genes with low expression may appear as zeros in the data. This phenomenon results in zero-inflated data, which is often considered problematic for analysis (Kharchenko *et al.* 2014). To alleviate this problem, we originally devised a weighting scheme that assigns lower weight to zeros in the data when solving regression problems. While this scheme improved performance with BEELINE’s curated datasets, it impaired performance with real RNA-seq data. In BEELINE’s simulations, dropouts are randomly introduced, whereas in reality, the dropout probability strongly depends on the gene’s expression level. Finally, we opted to not include the weighting scheme in the published method, and all results in the article are obtained without it. We also experimented with data smoothing by a *k*-nearest neighbour approach, but this did not improve performance either.

Regarding data normalization and transformation, GRIT does not differentiate between scales of different genes. Hence, it is not essential that all genes are on a similar scale. Log-transformation was found to perform well in latent structure discovery by Ahlmann-Eltze and Huber (2023). From the point of view of dynamical modelling, it is noteworthy that the square-root transformation conserves the functional form of linear degradation. That is, if $\frac{d}{dt}x(t)=-ax(t)$, then $\frac{d}{dt}\sqrt{x(t)}=-\frac{a}{2}\sqrt{x(t)}$. However, our test with the BEELINE RNA-seq data did not reveal a particularly strong effect of different transformations.

Possible extensions of this work include introducing growth and death rates of cell populations using unbalanced OT, or including cell-cell interactions (Almet *et al.* 2021) using an OT formulation allowing particle interaction (Ambrosio *et al.* 2005, Santambrogio 2015). Multi-omics integration would provide a thorough understanding of the regulatory relationships governing cellular mechanisms. For example, fast metabolite dynamics could be modelled using the framework of differential-algebraic equations based on a quasi-steady state approximation (Montanari *et al.* 2024). The so-called RNA-velocity approaches (La Manno *et al.* 2018, Qiu *et al.* 2022) enable estimation of derivatives for the gene expression vectors from the ratio of spliced and unspliced RNA (or using metabolic labeling). While these estimates are rather noisy, RNA-velocity could be integrated into an OT approach to leverage the benefits of both approaches. Indeed, the additional information in RNA-velocity could help with model inference, and conversely, the OT approach could help to reduce noise in the RNA-velocity. Zhang *et al.* (2021) propose such integration by introducing a cost function with two parts: the Euclidean norm and a cosine similarity between cell–cell difference and the RNA-velocity. In GRIT’s framework, such integration could be complemented by an additional cost (for the regression part) comprising terms $v-\frac{Ax}{∥Ax∥}$, where *v* is the (normalized) RNA-velocity of a cell with expression profile *x*.

## Supplementary Material

btaf394_Supplementary_Data

## Data Availability

The BEELINE benchmark data (Pratapa *et al.* 2020b) are available via Zenodo (doi: 10.5281/zenodo.3701939). The PINK1 (Novak *et al.* 2022) and LRRK2 (Walter *et al.* 2021) datasets are available via GEO with accession code for PINK1: GSE183248 and for LRRK2: GSE128040.
