# Supplementary material for: Dynamic gene regulatory network inference from single-cell data using optimal transport
Source: Bioinformatics. 2025 Jul 12;41(8):btaf394. doi: 10.1093/bioinformatics/btaf394 (PMC12352743; doi:10.1093/bioinformatics/btaf394)
Supplement: btaf394_Supplementary_Data [file btaf394_supplementary_data.pdf]

Supplementary material:

Dynamic gene regulatory network inference from single-cell data using optimal transport

François Lamoline [1], Isabel Haasler [2,3], Johan Karlsson [3], Jorge Gonçalves [1,4], and Atte Aalto [1,5,*]

[1] University of Luxembourg, Luxembourg Centre for Systems Biomedicine
[2]Department of Information Technology, Uppsala University
[3]KTH Royal Institute of Technology, Department of Mathematics
[4]University of Cambridge, Department of Plant Sciences
[5]Luxembourg Institute of Health, Department of Cancer Research

[*]Corresponding author, atte.aalto@uni.lu

Supplementary note 1: Method details

1.1 Model and setup

In this work, $\mathbb{R}^k$ represents the k-dimensional real space and $\mathbb{R}^{k\times m}$ the set of matrices of size $k \times m$. We denote by $\mathbb{1}_k$ the all-ones vector of $\mathbb{R}^k$ (though k is often omitted if it is clear from the context).

We model the cell population under investigation as a time-varying probability distribution $\mathbb{P}_t$ evolving according to the cellular physiology [13]. Due to the destructiveness of single-cell sequencing technologies, the data are considered as independent samples of distributions $\mathbb{P}_{T_k}$, $k \in \{0, \dots, N\}$, where $T_0, ..., T_N$ are the measurement times. Denote $\Delta T_k := T_k - T_{k-1}$. The matrices $Y_k \in \mathbb{R}^{n\times m_k}$ for $k = 0, ..., N$ contain the m_k samples (cells) measured at time T_k where n is the number of genes considered.

Cells are modelled as individuals evolving in the gene expression space. Mathematically, the gene expression levels of n genes form the state vector $x \in \mathbb{R}^n$ of a cell. It is assumed to be governed by the stochastic linear differential equation (SDE)

$$dx(t) = (Ax(t) + b)dt + \sqrt{\epsilon}dw(t), \qquad x_0 \sim \mathbb{P}_0 \tag{1}$$

where A is a sparse matrix, b is a constant load, w is a standard Brownian motion, and $\varepsilon > 0$ is the noise intensity. The initial state x_0 is assumed to be a realization drawn from a probability distribution $\mathbb{P}_0$. A comprehensive introduction and background material on stochastic differential equations can be found in [22]. Biologically, $x(t)$ represents the concentration of mRNA molecules, and A contains the regulatory parameters of the transcription factors (TFs). This means that the majority of the elements of the matrix A are in fact 0. This knowledge can be used to regularise the problem. Here, we restrict to additive noise in (1), which entails that the random fluctuations are assumed to be independent of the mRNA concentrations. Even though state-dependent noise is more realistic, it can lead to unidentifiable dynamics and overall a more complex problem to solve.

1.2 Optimal transport

The optimal transport (OT, sometimes called optimal mass transport) theory is a natural tool for finding a coupling between two probability distributions p and q and measuring the distance between them. It requires the definition of a cost function $c(x, y)$ (the transportation cost between x and y) between any samples $x \sim p$ and $y \sim q$. Optimal transport can be seen as a lifting of the cost function $c(x, y)$ between samples to a cost function between distributions p and q. The so-called Kantorovich formulation of the optimal transport problem is to find a joint distribution $\mu(x, y)$ that minimises the total cost of transportation [16] between the distributions p and q:

$$\begin{aligned} W(p, q) &:= \min_{\mu} \int c(x, y)\mu(x, y)dx\,dy \\ &\text{such that} \int \mu(x, y)dx = q(y) \quad \text{and} \quad \int \mu(x, y)dy = p(x). \end{aligned} \tag{2}$$

In the case $c(x, y) = d(x, y)^r$ where d is a metric, $W(p, q)^{1/r}$ is known as the Wasserstein distance. The solution of the optimal transport problem is the joint distribution $\mu(x, y)$, known as the transport plan — roughly interpreted as the amount of mass transported from x to y to minimise the total cost. It provides the coupling between the distributions.

In single-cell data, each time point consists of a point cloud of samples from a distribution. For comparison of point clouds, a discrete formulation of the OT problem is used. Say

$P = [p_1, ..., p_{m_P}] \in \mathbb{R}^{n \times m_P}$ is a matrix containing the points of the first set consisting of m_P points and $Q = [q_1, ..., q_{m_Q}] \in \mathbb{R}^{n \times m_Q}$ the second set consisting of m_Q points. As in the continuous case, a transportation cost needs to be defined between each pair (p_i, q_j) and these costs form a cost matrix $C \in \mathbb{R}_+^{m_P \times m_Q}$. These costs often arise from an underlying cost function, $C_{i,j} = c(p_i, q_j)$ defined on $\mathbb{R}^n \times \mathbb{R}^n$. The discrete formulation of the OT problem (with entropy regularisation) is

$$\begin{aligned} W(P, Q) := \min_M \sum_{i=1}^{m_P} \sum_{j=1}^{m_Q} \left[C_{i,j} M_{i,j} + \varepsilon M_{i,j} \log(M_{i,j}) \right] \\ \text{such that } M\mathbb{1} = \mu_P \quad \text{and} \quad M^\top \mathbb{1} = \mu_Q. \end{aligned} \tag{3}$$

where $\mu_P \in \mathbb{R}^{m_P}$ and $\mu_Q \in \mathbb{R}^{m_Q}$ are the weights of the samples in the respective distributions. Note that the discrete formulation arises directly from the continuous case by setting $p(x) = \sum_{j=1}^{m_P} \mu_{P,j} \delta_{p_j}(x)$ and $q(x) = \sum_{j=1}^{m_Q} \mu_{Q,j} \delta_{q_j}(x)$ in (2).

The discrete transport plan $M \in \mathbb{R}_+^{m_P \times m_Q}$ gives a coupling between the points in the two sets. The minimal value is used as a measure of the quality of fit between P and Q, but due to the entropy regularisation, $W(P, P) \neq 0$, meaning that it is not exactly a distance metric anymore. Below, the term *(entropy-regularised) optimal transport cost* is used for W. The entropy regularisation is fairly standard in OT problems and it has several functions [24, Chapter 4]. Firstly, it makes the problem mathematically well-posed (that is, the solution M depends continuously on the data P, Q). Secondly, it enables the use of the so-called Sinkhorn iterations to solve the problem efficiently [8]. Thirdly, it plays well along with the probabilistic interpretation of the OT problem (see Remark 1.1), introducing uncertainty to the OT problem corresponding exactly to the noise process $\varepsilon^{1/2} dw$ in (1).

The optimal transport cost is used to evaluate the performance of a dynamical system (1). The distribution measured at time T_{k-1} is first propagated through system (1). Then, this propagated distribution is compared with the population measured at T_k. To this end, consider a cell trajectory $x(t)$ satisfying (1) such that $x(T_{k-1}) = x_0$, where x_0 corresponds to a cell measured at time T_{k-1}. Due to linearity of the dynamics, and the Gaussianity of the noise process w, at time T_k, it holds that $x(T_k) \sim \mathcal{N}(m[x_0], G)$ where

$$\begin{aligned} m[x_0] &= e^{A\Delta T_k} x_0 + \int_0^{\Delta T_k} e^{As} b ds, \\ G &= \varepsilon \int_0^{\Delta T_k} e^{As} e^{A^\top s} ds. \end{aligned} \tag{4}$$

We wish to use the negative log-probability of a cell originating from x_0 (at time T_{k-1}) ending up at x_1 (at time T_k) as the transport cost from x_0 to x_1 (see Remark 1.1). This is given by

$$\begin{aligned} &-\log\left(p\big(x(T_k) = x_1 \,\big|\, x(T_{k-1}) = x_0\big)\right) \\ &\quad = \frac{1}{2}(x_1 - m[x_0])^\top G^{-1}(x_1 - m[x_0]) + \frac{1}{2}\log(\det G) + \frac{n}{2}\log(2\pi). \end{aligned}$$

The same cost can be obtained by arguments related to optimal control [4, 5], that is, minimizing the L^2-norm of a perturbation v in $\frac{d}{dt}x(t) = Ax(t) + b + v(t)$ when the initial and final states are fixed to $x(T_{k-1}) = x_0$ and $x(T_k) = x_1$. This duality between the linear-quadratic optimal control problem and the Gaussian maximum likelihood problem in the context of OT is also discussed in [12].

Unfortunately, the use of the exponential matrix in the cost function would eventually result in a complex optimisation problem for the estimation of A and b. To obtain a simpler optimisation problem and to improve robustness of the method, we instead consider a first-order

simplification of (4):

$$\begin{aligned} m[x_0] &\approx \big(I + \Delta T_k A\big)x_0 + \Delta T_k b \\ G &\approx \varepsilon \Delta T_k. \end{aligned} \tag{5}$$

In this simplification, G has become a scalar and it no longer depends on A. Therefore, the $\log(\det G)$ term (and the $n/2\log(2\pi)$ term) can be omitted from the cost. Moreover, the noise intensity ε is omitted from the cost as well (the reason becomes apparent in Remark 1.1 below), and thus we obtain the transport cost from x_0 to x_1:

$$\frac{1}{2\Delta T_k}\big\|x_1 - \big(I + \Delta T_k A\big)x_0 - \Delta T_k b\big\|^2. \tag{6}$$

To implement this cost in the transport problem (3), denote by $W(P,Q)$ the transport cost given by (3), where the underlying cost function is $c(p,q) = \frac{1}{2}\|p-q\|^2$. The comparison of data at time T_{k-1} propagated through the model and the data at time T_k is then done by calculating $\frac{1}{\Delta T_k} W\big((I + \Delta T_k A)Y_{k-1} + \Delta T_k b, Y_k\big)$. Note that to maintain the connection between noise intensity and entropy regularisation with this definition, $\varepsilon \Delta T_k$ should be used as the entropy regularisation. However, as explained below in Supplementary note 1.10, we estimate the entropy regularisation parameter directly from the data.

Remark 1.1. *As mentioned, the entropy-regularised transport cost is not a distance metric per se, since $W(P,P) \neq 0$. However, the entropy regularisation has an important role in handling noise in the observed distributions. The discrete OT problem (3) can be re-written as follows:*

$$\begin{aligned} \sum_{i,j}\big[C_{ij}M_{ij} + \varepsilon M_{ij}\log(M_{ij})\big] &= \varepsilon \sum_{i,j} M_{ij}\log(M_{ij}/\exp(-C_{ij}/\varepsilon)) \\ &= \varepsilon\, \mathrm{KL}\big(M \,\|\, \exp(-C/\varepsilon)\big) \end{aligned}$$

where $\exp(-C/\varepsilon)$ means elementwise exponentation. By definition (modulo the simplification (5)), the elements of the matrix $\exp(-C/\varepsilon)$ are (proportional to) the a priori probabilities of any cell x_0 from the population measured at time T_{k-1} ending up to the location of another cell x_1 from the population measured at time T_k. The OT problem can be interpreted as finding the joint distribution M that minimises the Kullback—Leibler divergence from $\exp(-C/\varepsilon)$ while satisfying the marginal conditions in (3), that is, matching with the observed distributions. This problem of matching an initial distribution with a target distribution under the assumption of some prior probabilities for the particle movements is known as the Schrödinger bridge problem [19, 6, 28].

As described in this section and the consistency theorem below, the entropy regularisation takes care of the additive noise in gene expression dynamics. It should be pointed out that additive noise with constant intensity is a simplifying assumption. Noise characteristics can have an effect on the quality of inference [7]. Within GRIT's framework, it would be possible to use gene-specific noise intensities by introducing a diagonal scaling matrix to the Euclidean norm in (6). However, multiplicative noise cannot be treated directly, but could be approximated by tuning gene-specific noise levels based on the genes' expression levels.

1.3 Model identification

The cost function can be defined for a known dynamical system, but the goal here is to use the OT approach in system identification, that is, determining the matrix A and the constant load vector b. We define the dynamical system as the solution to the minimisation problem

$$\min_{A,b} J(A,b) = \min_{A,b} \sum_{k=1}^{N} \frac{1}{\Delta T_k} W\big((I + \Delta T_k A)Y_{k-1} + \Delta T_k b, Y_k\big) + \|A\Lambda_A^{1/2}\|_F^2 + \lambda_b\|b\|^2 \tag{7}$$

where the diagonal matrix Λ_A and λ_b are regularisation parameters and W is the entropy-regularised optimal transport cost defined in (3) with $c(p,q) = \frac{1}{2}\|p-q\|^2$ as the cost function. Say matrix $C_k[A,b] \in \mathbb{R}^{m_{k-1}\times m_k}$ contains the costs as defined in the previous section for measurements in Y_{k-1} and Y_k. That is, the element (i,j) of this matrix is obtained by (6) where x_0 is the ith column of Y_{k-1} and x_1 is the jth column of Y_k. Then expanding the definition of the optimal transport cost W results in a combined optimal transport and system identification problem:

$$\min_{A,b,M_k} \sum_{k=1}^{N} \sum_{i,j} \left[[C_k[A,b]]_{i,j}[M_k]_{i,j} + \varepsilon [M_k]_{i,j} \log [M_k]_{i,j} \right] + \|A\Lambda_A^{1/2}\|_F^2 + \lambda_b \|b\|^2 \tag{8}$$

subject to $M_k\mathbb{1} = \mathbb{1}$ and $M_k^\top \mathbb{1} = \mu_k$. With this definition of the marginal distributions, each propagated cell is assumed to have mass one, whereas the masses of the cells in the target distribution are scaled. Usually the scaling is simply $\mu_k = \frac{m_{k-1}}{m_k}\mathbb{1}$, except in the cases with branching dynamics (see Supplementary note 1.7). The combined cost function is strongly convex (quadratic) with respect to A and b when the transport plans M_k are fixed. Conversely, when A, b are fixed, the problem is convex (in fact strongly convex thanks to the entropy regularisation) with respect to the transport plans M_k. Unfortunately, however, the componentwise convexity does not imply joint convexity. Indeed, the cost function in (8) may have several local minima. The componentwise convexity property nevertheless motivates a coordinate-descent type algorithm for solving the problem. Since the cost function is quadratic for the variables A and b, it can be solved analytically for fixed M_k. When A, b are, in turn, fixed, (8) becomes a standard entropy-regularised OT problem and it can be solved efficiently with Sinkhorn iterations. The full problem is solved by alternating between these steps until convergence.The estimation of the entropy regularisation parameter ε and the regression regularisation parameters Λ_A and λ_b is explained below in Supplementary note 1.10.

Note that with the transport plans M_k fixed, the regression problem (8) has $\sum_{k=1}^{N} m_{k-1}m_k$ (degenerate) data points. As shown below in Supplementary note 1.11, the problem can be reduced into a smaller and better explicable regression problem by defining a target point at time T_k for each cell $x_i(k-1)$ measured at time T_{k-1} as a weighted average of the cells measured at time T_k. The weights are given by the ith row of the transport plan M_k (that sums up to one due to the marginal constraint $M_k\mathbb{1} = \mathbb{1}$). With matrix notation, the target points for all cells in the matrix Y_{k-1} are given by $Y_k M_k^\top$. Moreover, these target points can be used to estimate derivatives for the cells (corresponding to RNA velocity [18]) by a difference quotient. Define a matrix containing all measured cells from time points T_0 to T_{N-1} — augmented by a row of ones to include b in the same regression

$$Y = \begin{bmatrix} Y_0, \ldots, Y_{N-1} \\ \mathbb{1}^\top \end{bmatrix}. \tag{9}$$

Their estimated derivatives are then given by

$$Z = \left[\frac{1}{\Delta T_1}\left(Y_1 M_1^\top - Y_0\right), \ldots, \frac{1}{\Delta T_N}\left(Y_N M_N^\top - Y_{N-1}\right) \right]. \tag{10}$$

Then the augmented matrix $[A, b]$ minimising (8) can be solved row-by-row from linear regression problems

$$\min \|Z_i - [A_i, b_i]Y\|_D^2 + \|\Lambda_A^{1/2} A_i^\top\|^2 + \lambda_b b_i^2, \qquad i = 1, \ldots, n, \tag{11}$$

where Z_i and A_i are the ith rows of matrices Z and A, and D is a diagonal matrix with diagonal elements $[\Delta T_1 \mathbb{1}_{m_0}^\top, \ldots, \Delta T_N \mathbb{1}_{m_{N-1}}^\top] \in \mathbb{R}^{m_0+\ldots+m_{N-1}}$ arising from the scaling by ΔT_k in (7). Note that the regression problem (11) has $\sum_{k=1}^{N} m_{k-1}$ data points instead of $\sum_{k=1}^{N} m_{k-1}m_k$ of the original problem (8).

To reduce the effect of noise in the data, for the solution of the OT problem, the dimension of the space is reduced by means of principal component (PC) reduction. The dimension of the PC space is $0.9n$ (rounded) but at most 100. Note, however, that this dimension reduction is applied only in the calculation of the costs in (6), but the identification of the dynamical system is done in the full n-dimensional space.

1.4 Gene regulatory network inference

The variable selection step used for GRN inference is based on a greedy forward-backward sweep (inspired by [30] to which we also refer for details on the greedy approach) applied on the regression problem (11) one target gene at a time. As discussed in [30], the forward greedy approach may make mistakes that are not corrected and a backward greedy algorithm is more likely to work better. However, in high-dimensional problems, the backward algorithm may fail, if all possible regressors are allowed to be active at the same time. To initialise the backward greedy algorithm, the initial set of regressors is selected based on a combination of gene-gene correlations and a forward greedy algorithm. First, the regulators with high (positive or negative) correlation with the target gene are chosen in the regressor set (top $N_{\text{reg}} - 5$ genes), including the target gene itself, and the constant load term b_i. To complement the set, a forward greedy algorithm is applied. At every step, the regressor yielding a highest decrease in the cost function is added to the regressor set. The forward sweep is continued until N_{reg} regressors are included. The backward greedy algorithm is then carried out, at every step removing the regressor that yields the smallest increase to the cost function. The exceptions are the gene itself and the constant load terms that are always kept in the regressor set. The backward greedy steps are continued until only these fixed regressors remain.

The method output is a confidence score for each link. During the backward phase, when there are p active regressors (not including the constant load terms), the cost function increments are collected in a vector $\Delta J \in \mathbb{R}^p$ which is then normalised by dividing with $\max \Delta J$. Then, the link whose removal would yield the highest cost function increase, has value 1 in this vector. The final score of a particular link is the average of these scaled cost function increments calculated over those regressor sets where the link was active. Notice that with this scoring scheme, those links that at are never included in the active regressor set will get score zero. This scoring scheme has been developed with the aim to have values between zero and one for all genes, and to have values that are not too dispersed.

Note that for problems with small enough dimension, $n \leq N_{\text{reg}}$, only the backward greedy algorithm is applied starting from a full regressor set.

1.5 Inference of perturbation targets

Perturbations are dealt with by introducing an additional vector b into the dynamics (1) in the variable selection phase of the method, but only to the dataset with the perturbation. That is, a line is added to the concatenated matrix Y defined in (9). This line has zeros in positions corresponding to the control experiment, and ones in positions corresponding to the perturbation experiment. This term is then considered as a possible regulator exactly as any gene, yielding an additional column into the method output matrix of confidence values of link existence. Perturbation target inference is validated in Supplementary note 2.1.

1.6 Inference of mutation effects

Mutations are handled by introducing a new line into the concatenated matrix Y defined in (9). This line has zeros in positions corresponding to the control experiment, and the expression values of the mutated gene in positions corresponding to the mutation experiment. Unlike with perturbations, this line is not used as an independent candidate regulator, but instead, it is considered an active regressor whenever the mutated gene is active. Effectively this means that the column of the A-matrix corresponding to the control and mutation datasets are allowed to be different. The introduction of a new row in the Y matrix also means that a new column is introduced in the A-matrix. This additional column is the difference of the A-matrix column corresponding to the mutation dataset compared to the control dataset. The backward greedy phase of the variable selection is then complemented with an additional step for evaluating mutation targets. If the mutated gene is an active regressor, the mutation is scored by calculating the cost function increment for removing only the additional mutation-row from the active regressor set (note that the mutated gene itself is scored by removing both the row corresponding to the mutated gene, and the additional row with zeros in places corresponding to the control experiment). This cost increment is then augmented into the ΔJ vector and the method then proceeds as described in Supplementary note 1.4. This results in an additional column in the method output matrix of confidence values of link existence. Mutation effect inference is validated in Supplementary note 2.2.

1.7 Accounting for branching dynamics

Branching dynamics are a profoundly nonlinear phenomenon, and a linear model class is not ideal for dealing with it. A stable linear system (1) can only have one steady state, given by $x = -A^{-1}b$. To include branching dynamics and enable multiple steady states, different branches are assigned to different constant vectors b in (1). Moreover, branching is taken into account in the OT problem by adjusting the weights of the cells μ_k and the costs C_k in (8). The goal is to discourage mass transport between branches in the solution of the OT problem.

The adjustment of the weights is done as follows. Say each measurement matrix Y_k is accompanied by a matrix $B_k \in \{0,1\}^{N_B \times m_k}$ where N_B is the number of branches. This is an indicator matrix whose columns contain $\{0,1\}$-valued variables indicating to which branch(es) the corresponding cell belongs to. Denote by $m_b(k)$ the sum of the bth row of the matrix B_k. Then, define $\tilde{B}_k$ as the normalised indicator matrix, where each column of B_k is divided by the column sum. The idea is that cells belonging to multiple branches have their mass (in the sense of the OT problem) equally divided between branches. In case cells are only allowed to belong to one branch, it holds that $\tilde{B}_k = B_k$. Denote by $\tilde{m}_b(k)$ the sum of the bth row of $\tilde{B}_k$. Then the mass distribution μ_k of the cells of the kth time point for the OT problem (3) is given by the column sum of $\text{diag}\big(\{m_b(k-1)/\tilde{m}_b(k)\}_{b=1}^{N_B}\big)\tilde{B}_k$.

This scaling is done to enable the existence of a solution M_k that does not mix cells between branches. To encourage such solution, the cost matrix entries $[C_k[A,b]]_{i,j}$ are scaled by a factor higher than one when cells i and j do not belong to the same branch. This factor has a default value 2, but it can be changed by the user. To identify cell pairs between two time points that do not belong to the same branch, it suffices to find zeros of the matrix $B_{k-1}^{\top}B_k \in \mathbb{R}^{m_{k-1} \times m_k}$.

Note that in this setup, one cell can belong to multiple branches (e.g., cells before the branching has taken place), although in the output of Slingshot, each cell is assigned to exactly one branch.

1.8 Lineage tracing and branch labeling

The optimal transport maps M_k obtained from (8) can be used to reconstruct cell trajectories, and to identify ancestor and descendant cells across measurement times. The columns (scaled to sum up to one) of the transport plan matrix M_k contain information about the propagated ancestors, while the rows (scaled) tell about the propagated descendants. It is possible to give a probabilistic interpretation to the scaled rows and columns of the transport matrix. That is, $[M_k]_{i,j}/\sum_{l=1}^{m_k}[M_k]_{i,l}$ gives the probability that cell i measured at time T_{k-1} would be the ancestor of cell j measured at time T_k, and $[M_k]_{i,j}/\sum_{l=1}^{m_{k-1}}[M_k]_{l,i}$ gives the probability that cell j would be the descendant of cell i. To identify descendant and ancestor cells across multiple time transitions, one can simply multiply the transport matrices corresponding to the transitions.

Among other things, the lineage tracing can be used to reconstruct branch labels that improve the performance of our method. This requires clustering the cells measured at the final time point where typically the clusters/branches can be identified relatively easily. We provide a function to then reconstruct branch labels for all time points using the transport matrices obtained from the method applied without using branch labels. The cluster labels should be given in the same format as the branch labels B_k described in Supplementary note 1.7, that is, the label matrix is $C \in \{0,1\}^{N_B \times m_N}$ where N_B is the number of clusters/branches and m_N is the number of cells in the final time point. This matrix should contain exactly one non-zero entry on each column.

The branch label for the last time point will be directly the cluster indicator given by the user, $B_N = C$. To reconstruct branch labels for a time point $k < N$, we calculate the transport map across multiple time points as a product $\hat{\mathbf{T}}_{k\to N} := \prod_{i=k+1}^{N} M_i$ which is then normalised by the row sum to get $[\mathbf{T}_{k\to N}]_{i,j} := [\hat{\mathbf{T}}_{k\to N}]_{i,j}/\sum_{l=1}^{m_N}[\hat{\mathbf{T}}_{k\to N}]_{i,l}$. Each column of the matrix $C\hat{\mathbf{T}}_{k\to N}^\top \in [0,1]^{N_B,m_k}$ sums up to one. A column corresponds to a cell measured at time T_k, and it gives the percentage of the descendants of the cell belonging to each branch (recall that the OT approach can split the cells such that each cell is associated with several past and future cells). The branch label matrix B_k is then constructed by inserting a 1 on each position where the corresponding entry of the matrix $C\hat{\mathbf{T}}_{k\to N}^\top$ is greater than a threshold value, for which the default value is $1/(2N_B)$. Note that with this approach, a cell can belong to multiple branches. If the user sets a threshold higher than $1/N_B$, it is possible that on some columns, no entry is higher than the threshold. In such case, a 1 is inserted in the column on the position with the highest entry to ensure that every cell is assigned to a branch. This approach is validated in Supplementary note 2.3 by comparing the branch assignments with those given by Slingshot.

1.9 Additional features

Several experimental datasets can be combined into one inference problem. This, however, should only be done when the experiments are carried out on isogenic organisms.

While the link scoring described above always produces positive confidence values for link existence, the signs of the entries of the inferred A-matrix can be used to determine whether a regulatory link is an activation or an inhibition. GRIT can be requested to produce a signed list of confidence scores.

Parallelised computing is readily built-in in GRIT. The optimal transport problems are always solved independently for each time transition $T_{k-1} \to T_k$, and therefore this step can be straightforwardly parallelised to up to N processors. The model update step, in turn, as well as the variable selection are done independently one target gene at a time, which can be done in parallel processes.

1.10 Parameters

GRIT has few tuning parameters. As discussed above, the coefficient for entropic regularisation ε has a direct interpretation in terms of noise intensity in the data. However, noise intensity is not known, and, moreover, the real data are anyway not produced by linear discrete-time dynamics. The parameter is therefore selected to ensure robust solvability and stability of the Sinkhorn iterations used to solve the OT problem. Each time the OT problem is solved, as a preliminary step, the median of all entries in the cost matrix is calculated and $\varepsilon_0\text{median}(C)$ is used as the regularisation parameter. The coefficient ε_0 has a default value 0.05, but it can be adjusted by the user. Note that this scheme leads to different regularisation parameters for different time steps.

The Tikhonov regularisation parameters Λ_A and λ_b in (7) are defined with the help of the matrix Y defined in (9) by calculating $\text{diag}(YY^\top)$, dividing by the total number of cells, and multiplying by the experiment time range $T_N - T_0$ (or the sum of time ranges in case multiple experiments are concatenated). The resulting vector has dimension $n + N_B$. The first n components multiplied by a parameter C_{reg} are the diagonal entries of the matrix Λ_A. The remaining N_B elements multiplied by $10C_{\text{reg}}$ are used as the regularisation parameters for their respective b vectors corresponding to different branches. By default, $C_{\text{reg}} = 0.01$ is used, for which sensitivity analysis is presented in Supplementary figure 11. This regularisation is invariant to the time scale (results do not change if time unit is changed from days to hours, for example) and to the scales of different genes. Overall, the regularisation is rather mild, but it ensures stability of the solution even for low cell numbers and high dimension.

1.11 Reduction of the regression problem

The optimisation problem formulated in (8) is a quadratic problem with respect to A, b when the transport plans M_k are fixed. To solve the quadratic problem, let us calculate the derivative of one entry in the cost matrix (using again x_0 and x_1 as generic vectors as in (4)–(6))

$$\frac{d}{dA}C_k[A,b] = \frac{d}{dA}\frac{1}{2\Delta T_k}\left\|x_1 - \big(I + \Delta T_k A\big)x_0 - \Delta T_k b\right\|^2 = -\big(x_1 - \big(I + \Delta T_k A\big)x_0 - \Delta T_k b\big)x_0^\top.$$

Then, denoting by $x_j(k)$ the cell j measured at time T_k, it holds that

$$\begin{aligned}
&\frac{d}{dA}\sum_{i=1}^{m_{k-1}}\sum_{j=1}^{m_k}[C_k[A,b]]_{i,j}[M_k]_{i,j} \\
&= -\sum_{i=1}^{m_{k-1}}\sum_{j=1}^{m_k}[M_k]_{i,j}\big(x_j(k) - \big(I + \Delta T_k A\big)x_i(k-1) - \Delta T_k b\big)x_i(k-1)^\top.
\end{aligned}$$

Here anything multiplying A does not depend on j, and therefore the marginal condition $M_k\mathbb{1} = \mathbb{1}$ gives

$$\begin{aligned}
&\sum_{j=1}^{m_k}[M_k]_{i,j}\big[\big(I + \Delta T_k A\big)x_i(k-1) + \Delta T_k b\big]x_i(k-1)^\top \\
&= \big[\big(I + \Delta T_k A\big)x_i(k-1) + \Delta T_k b\big]x_i(k-1)^\top
\end{aligned}$$

and therefore

$$\frac{d}{dA}\sum_{i=1}^{m_{k-1}}\sum_{j=1}^{m_k}[C_k[A,b]]_{i,j}[M_k]_{i,j}$$
$$= -\Delta T_k \sum_{i=1}^{m_{k-1}} \left[\frac{1}{\Delta T_k}\left(\sum_{j=1}^{m_k}[M_k]_{i,j}x_j(k) - x_i(k-1)\right) - Ax_i(k-1) - b\right] x_i(k-1)^\top.$$

Similar calculation can be done for the derivative with respect to b.

Basically this means that when solving the optimal A and b, this is equivalent to a regression problem where for each cell $x_i(k-1)$ measured at time T_{k-1}, a target point at time T_k is defined as a weighted average of the cells measured at time T_k, where the weights are given by the ith row of the transport plan M_k. Moreover, this target point can be used to estimate a derivative for $x_i(k-1)$ by a difference quotient. That is, define a matrix containing all measured cells from time points T_0 to T_{N-1} (augmented by a row of ones)

$$Y = \begin{bmatrix} Y_0, \dots, Y_{N-1} \\ \mathbb{1}^\top \end{bmatrix}$$

and a matrix containing their estimated derivatives

$$Z = \left[\frac{1}{\Delta T_1}\left(Y_1 M_1^\top - Y_0\right), \dots, \frac{1}{\Delta T_N}\left(Y_N M_N^\top - Y_{N-1}\right)\right].$$

Then the augmented matrix $[A, b]$ can be solved row-by-row from linear regression problems $\min \|Z_i - [A_i, b_i]Y\|_D^2$ for $i = 1, ..., n$, where Z_i and A_i are the ith rows of matrices Z and A, and D is a diagonal matrix with diagonal elements $[\Delta T_1 \mathbb{1}_{m_0}^\top, \dots, \Delta T_N \mathbb{1}_{m_{N-1}}^\top] \in \mathbb{R}^{m_0 + \dots + m_{N-1}}$.

Supplementary note 2: Additional results

2.1 Perturbation target inference

A standout feature of a differential equation based method is the ability to deal with perturbations in a straightforward manner. Given data on a control experiment and a perturbation experiment, GRIT can infer targets of the perturbation (for example, a drug). Perturbation target inference by GRIT is explained in Supplementary note 1.5. Essentially, an additional vector b is added to (1) that is active only in the perturbation dataset.

To validate the inference of targets of an external perturbation, data were generated from the modified nonlinear system presented in Supplementary note 5.4. In the modification, the basal transcription rates of different genes were modulated. Six cases were generated where either one, two, or three genes were affected by the perturbation with strength of either 30% or 60% increase in the basal transcription rate. Each case consisted of 20 replicates with a control experiment simulated from the model without any perturbations, and a perturbation experiment. The perturbation targets were randomly chosen in each replicate.

GRIT is given the data for both the control and perturbation experiments simultaneously and it is informed that the second experiment has a perturbation. GRIT then outputs an additional vector consisting of confidence values on which genes' dynamics are likely directly affected by the perturbation. These vectors were concatenated for all replicates into one 10×20 matrix, that could be compared to a ground truth matrix. The results for the four cases are shown in Supplementary figure 1a. To provide a reference to compare with, we also included results obtained by calculating gene-gene correlations separately for the control and perturbation experiments, and summing up the absolute differences of correlations over each row of the correlation matrix. These differences should pinpoint changes in gene dynamics. GRIT's perturbation target inference far exceeds the performance of the correlation differences.

2.2 Mutation effect inference

GRIT can be used to identify regulations that are altered due to a mutation in a known gene. As with perturbation target inference, a control dataset and a mutation dataset are needed, that should otherwise correspond to the same experimental conditions, and preferably should be isogenic except for the mutation. Such datasets can be generated by gene editing that allows inflicting targeted mutations into a cell line. The mutation effect inference by GRIT is explained in Supplementary note 1.6. Essentially, the column in the matrix A corresponding to the mutated gene is allowed to differ between the control and mutation datasets.

To validate the inference of affected regulations due to a mutation, data were generated from the modified nonlinear system (details in Supplementary note 5.4). In the modification, gene 9 directly regulates three other genes. Four cases were generated where either one or two of the regulations from gene 9 were modulated by a coefficient 0.5 or 0.8 corresponding to a 50% or 20% loss of function for the corresponding regulations. Each case consisted of 30 replicates with a control experiment simulated from the model without any perturbations, and a mutation experiment. In the 30 replicates of each case, each gene or each two gene combination was modulated in 10 replicates.

As with the perturbation target inference, GRIT is given both the control and mutation experiments simultaneously and it is informed of the mutation of gene 9 in the second experiment. GRIT outputs an additional vector consisting of confidence values on which genes' dynamics are likely affected by the mutation. These vectors were concatenated for all replicates into one 10×30 matrix, that could be compared to a ground truth matrix. The results for the

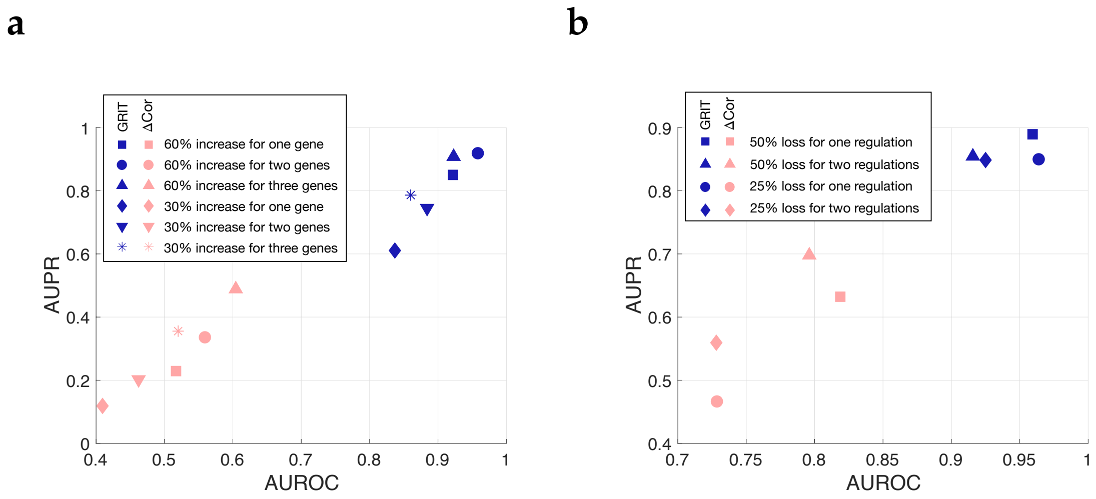

Supplementary figure 1: **a:** Results for the inference of perturbation targets by GRIT and by differences in gene-gene correlations in the six different experiments. **b:** Results for the inference of mutation effects by GRIT and by differences in gene-gene correlations in the four different experiments.

four cases are shown in Supplementary figure 1b. As with the perturbation experiment, we also included reference results obtained by calculating gene-gene correlations separately for the control and mutation experiments, and looking at the absolute differences of correlations corresponding to the mutated gene 9 between the two experiments. Again, GRIT's mutation effect inference performs much better in the task than the correlation differences. It should be noted, however, that the mutation target inference method requires the knowledge of the mutated gene(s). If this is not the case, perturbation target inference should be used instead.

2.3 Lineage tracing by transport plans

Reaching a complete understanding of a differentiation process or disease progression requires the ability to analyse cell paths. However, due to the destructive nature of the single-cell measurements, cell paths cannot be directly observed. The transport plans M_k can be used for lineage tracing, in particular, for probabilistic identification of ancestor and descendant cells.

In Supplementary note 1.8, we introduced a scheme that constructs branch labels by tracing the ancestor lineage of cell clusters defined on the final measured time point. More specifically, GRIT is first applied without the branch labels, then cells measured on the final time point are clustered, and finally branch labels are constructed by identifying cells at earlier time points whose descendants belong to the identified clusters of the final time point. This branch labeling scheme is illustrated here on the simulated data of the system producing trifurcating behavior in the BEELINE benchmark. In this dataset, the initial population is unimodally distributed, and over time the population splits into three subpopulations. Branch indicators obtained by Slingshot are provided with the data (three branches corresponding to the three subpopulations). The data are divided into five time points. The GRIT branch labeling scheme was run by using the provided Slingshot branch labels for the final time point T_4 as the cluster labels. The branch labels from GRIT for time points T_k, $k = 0, 1, 2, 3$, can then be compared to the Slingshot branch labels. The confusion matrices for different time points are shown in Supplementary figure 2. It can be noted that the identified ancestors respect the branch assignment by Slingshot very well, except for the time point T_0. This, however, is not surprising since the

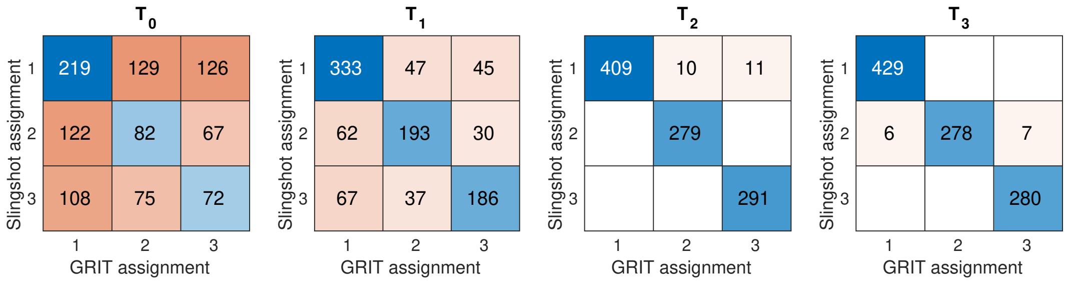

Supplementary figure 2: Confusion matrices for branch assignments by GRIT compared to branch assignments of Slingshot at different time points T_0 to T_3. Note that branch assignments from Slingshot at time T_4 were used as cluster labels for GRIT to ensure a meaningful comparison, and therefore a confusion matrix for T_4 is not shown.

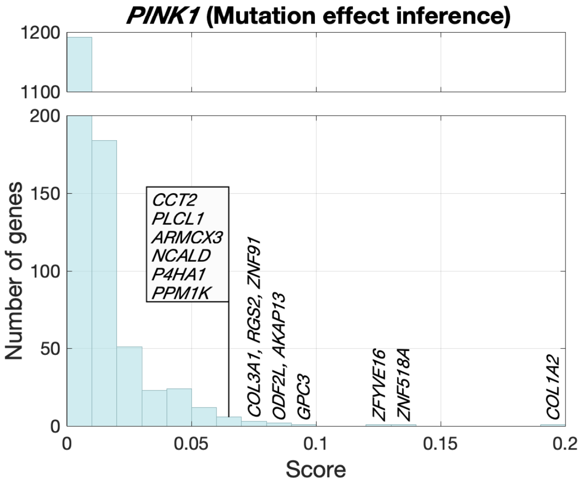

Supplementary figure 3: Histogram of the mutation effect scores with high-confidence genes indicated in the *PINK1* case.

initial distribution is unimodal, and therefore the branch assignments are arbitrary. One should also keep in mind that Slingshot branch assignments are by no means a "ground truth", but the result simply demonstrates a high level of agreement between the two methods.

2.4 Mutation effect inference for the *PINK1* dataset

The mutation effect inference can only detect genes to which a link from *PINK1* has been inferred, and therefore only a handful of genes get a score that is clearly non-zero. Therefore, these results did not yield any significantly enriched pathways (when corrected for multiple hypothesis testing), except for one term with dimension 1500. Enrichment results for the mutation effect inference are therefore not shown. As it is not possible to evaluate the soundness of the results obtained by the mutation effect inference by inspecting the results of the enrichment analysis, the results are only shown in the supplementary material.

Supplementary figure 3 shows the histograms of gene scores for being mutation targets and lists the most highly scoring genes. Of the high-scoring genes in the *PINK1* case with mutation effect inference, *RGS2* has been identified as a regulator of *LRRK2* and neuronal toxicity [9]. *GPC3* interacts with *DJ-1* (*PARK7*) [21].

Supplementary note 3: Details on method application and result processing

3.1 Datasets, preprocessing, and details of method application

Method validation is done using the BEELINE benchmarking pipeline [26, 25]. The pipeline consists of three different types of datasets. The *synthetic* dataset consists of simulated data from six systems that are purpose-designed to reproduce certain qualitative behaviours. For each system, ten datasets are simulated, and subsets with five different numbers of cells (100, 200, 500, 2000, or 5000) are provided for the inference tasks. This results in 300 tasks in total. The cases with 2000 and 5000 cells are used in the main comparison and the results for other cases are presented in supplementary material. The *curated* dataset consists of simulated data from four models from literature. Again, ten sets are simulated for each model, and for each of these replicates, three sets are created with different rates of dropouts in the data (dropout rates of 0%, 50%, and 70%). This results in 120 inference tasks in total. The case without dropouts is used in the main comparison and the results for other cases are presented in supplementary material. Finally, five real single cell RNA-Seq datasets are used for validation. One of the datasets corresponds to cell differentiation, and there are three different target cell types. The branching dynamics are identified with Slingshot, and the branches are treated as separate inference tasks. For each case, four different ways to select the genes of interest are used, resulting in 28 inference tasks in total.

GRIT requires data in batches of cells measured at different times, as is typical for a single-cell experiment. The simulated data of BEELINE do not follow this paradigm, but instead consists of cells whose simulation time has been randomly selected from some interval. We generated measured batches by ordering cells by their pseudotime (pseudotime by Slingshot is provided with the BEELINE data [25]), and then divided the data into eight or fifteen separate time points. The measurement time for each batch was the average of the pseudotimes of the cells in the batch. The real data in BEELINE consist of separate time points, except for three cases. In these three cases, we used the pseudotime (provided together with the data in [25]) to split the data into six time points.

Regarding the final experiment on Parkinson's disease (PD) associated mutations, the *LRRK2* dataset monitored human patient specific induced pluripotent stem cell (iPSC)-derived neuroepithelial stem cells' (hNESCs) differentiation into DA neurons [29]. The mutant cells had the PD-associated *LRRK2*-G2019S mutation while the control cells were isogenic, corrected for the *LRRK2* mutation, and the samples were analysed on days 0, 10, 14, and 42 of the differentiation processes. The *PINK1* dataset monitored human patient specific induced pluripotent stem cells' (iPSCs) differentiation into DA neurons [21]. The mutant cells had the PD-associated *PINK1*-I368N mutation while the control cells were from age-sex matched controls, and the samples were analysed on days 0, 6, 15, and 21 of the differentiation processes, and on an additional day 10 for the control cells.

For the *LRRK2* data, cells with less than 1000 reads were filtered out and only genes included in all data files (time points) were kept. In the *PINK1* dataset, the released data have fewer cells overall but the quality seems better, as all cells have more than 1000 reads, except for time points 10 and 21 in the control case. In fact, time point 10 has average read count 2804 and time point 21 has 1292 compared to the average 12717 for the remaining three time points. Due to this drastic discrepancy, these two time points are excluded from the analysis. Then, genes that were expressed in less than 10% of the cells were filtered out. The resulting *LRRK2* dataset had 6507 genes (down from 16662) with 3838 cells in total for the control case and 4403 cells in

the mutant case. The filtered *PINK1* dataset had 8756 genes (down from 18097) with 2518 cells in the control case and 1977 cells in the mutation case. Wasserstein distances were calculated individually for all 1D distributions of different genes between consecutive time points (in 1D, the Wasserstein distance is the L^1 norm between the cumulative distribution functions) and added up over all time points and both conditions. The genes were then sorted based on this dynamical variability, and 500, 1000, 1500, or 2000 most highly varying genes were given to GRIT, in the first stage, and dimensions 750 and 1250 were later included for the *LRRK2* case and dimensions 1250 and 1750 for the *PINK1* case (see Supplementary note 3.3 for details). In the *LRRK2* dataset, *LRRK2* itself is only expressed in 49 control cells and 115 mutant cells, and mostly in the final time point on day 42, and it is filtered out in the first phase. It can hardly be expected that any differences in regulations by *LRRK2* could be inferred based on a handful of reads. This experiment is therefore treated as a perturbation dataset rather than a mutation dataset, and it is treated as described in Supplementary note 1.5. *PINK1* is better expressed, but it is not among the 2000 most highly varying genes. Therefore *PINK1* is manually added to the list of genes after the dimension reduction based on gene variability. GRIT was then run in two ways, regarding the second experiment either as a mutation experiment (treated as described in Supplementary note 1.6), or as a perturbation experiment (treated as described in Supplementary note 1.5). Both results are reported.

3.2 Performance evaluation

Standard scores are used for evaluation, namely the Area Under the Receiver Operating Characteristics curve (AUROC), and the Area Under the Precision–Recall curve (AUPR). For a straightforward comparison, in the context of the BEELINE benchmarking pipeline, we use the performance metrics proposed in BEELINE. Since the AUPR values depend on the sparsity level of the network, the AUPR is used as a ratio of the AUPR of the inference results and the expected AUPR for random matrix. Then, AUPR-ratio 1 means a result comparable to random guessing. An additional metric is the Early Precision Ratio (EPR), which is also used in BEELINE. To calculate the EPR, k links with highest confidence assigned by a method are checked, and the share of true positive links is calculated. This share is then divided by the sparsity level of the network. This scaling means that an EPR of 1 corresponds to performance equivalent of random guessing. The value of k is chosen as the number of links in the ground truth network.

The summary results for the BEELINE benchmark shown in the main text are based on ranking the methods in each task. For synthetic dataset, AUPR-ratios for the six systems are used for this ranking (corresponding to [26, Fig. 2]). For the curated dataset, ranks were separately determined using the AUPR-ratio and the EPR (corresponding to [26, Fig. 4]). For the RNA-Seq data, EPRs for each seven tasks were used for ranking with the STRING and Non-specific ChIP-Seq networks used as ground truths in the cases TFs + 500 and 1000 genes (corresponding to [26, Fig. 5]). The results obtained using the Celltype-specific ChIP-Seq as the ground truth (and the lof/gof network for mESC) seemed to bear almost no correlation with the predictions of any of the evaluated methods, and these were therefore excluded from the summary results. The summary results for the RNA-Seq data are then based on 28 ranks (7 tasks, two different sizes, and two different ground truths).

3.3 Pathway enrichment analysis for the *PINK1* and *LRRK2* results

As described above, for the analysis of the *PINK1* and *LRRK2* datasets, subsets of genes were first selected based on their dynamical variability, and then GRIT was run on these subsets.

Of particular interest in the results are the genes that GRIT identifies as potential direct downstream effects of the mutation. GRIT outputs a scored list of all genes in the analysis where the score indicates the confidence for the gene being a perturbation target. To gain insight into these results, pathway enrichment analysis is then applied to this list.

To avoid bias due to the selection of genes included in the analysis and to avoid the need to select a threshold for the results, the following approach was taken. The pathways in the KEGG database [15] were used in the enrichment analysis. First, we determined the intersections of all terms in the KEGG database and the set of genes included in the analysis (using the g:profiler tool [17]). The genes included in the analysis were ordered by the confidence score for being a perturbation target according to GRIT. We determined the ranks of all genes for each KEGG term on the ordered list. Then, a Kolmogorov–Smirnov (KS) test was performed with the null hypothesis that these ranks were uniformly distributed. One-tail KS test was used with the alternative hypothesis that the ranks originate from a cumulative distribution that is greater than the cumulative distribution of the uniform distribution to identify only pathways that are enriched in the high score genes. To correct for multiple hypothesis testing, p-values were adjusted by the Benjamini–Hochberg false discovery rate approach [3].

The enrichment results were also used to choose which results to report among the different number of genes included in the analysis as described above in Supplementary note 3.1. Initially, when more genes are included in the analysis, the statistical significance of the enrichment results increases, but later it may drop when too many genes are added, potentially due to increasingly difficult identification in higher dimension. For each tested dimension, we counted the number of significantly enriched terms (adjusted p-value below 0.05), and the results with the highest number of enriched terms are reported. From the initial tested dimensions (500, 1000, 1500, 2000), the highest number of enriched terms was obtained with 1000 for *LRRK2* and for 1500 for *PINK1*. Then, also dimensions 750 and 1250 were tested for *LRRK2* and dimensions 1250 and 1750 for *PINK1*, but these did not yield more enriched terms. Therefore, results for dimension 1000 are reported for *LRRK2* and 1500 for *PINK1*.

Supplementary note 4: Consistency theorem for the method

In the absence of branching dynamics, and with m cells measured at each time point, the method is based on minimisation of the cost function

$$\min_{A\in\mathbb{R}^{n\times n},b\in\mathbb{R}^n}\sum_{k=1}^{N}\frac{1}{2\Delta T_k}W\big((I+\Delta T_k A)Y_{k-1}+\Delta T_k b, Y_k\big)+\frac{1}{m}\|A\Lambda_A^{1/2}\|_F^2+\frac{1}{m}\lambda_b\|b\|^2 \tag{12}$$

where $W(P,Q)$ is the discrete optimal entropy-regularised transport cost defined between point clouds $P=[p_1,...,p_{m_P}]$ and $Q=[q_1,...,q_{m_Q}]$ by

$$W(P,Q)=\min_{M\in\mathbb{R}^{m_P\times m_Q}}\sum_{i=1}^{m_P}\sum_{j=1}^{m_Q}\left[M_{i,j}\|p_i-q_j\|^2+2\varepsilon M_{i,j}\log(M_{i,j})\right]+2\varepsilon\log(m_P m_Q) \tag{13}$$

subject to $M\mathbb{1}=\frac{1}{m_P}\mathbb{1}$ and $M^\top\mathbb{1}=\frac{1}{m_Q}\mathbb{1}$. Note that here the marginal constraints are slightly different from the main text. The different definition is compensated by scaling by $1/m$ in (12), which makes cost function (12) equivalent to the cost function (7) (up to multiplication by $1/m$ to prevent the cost from tending to infinity as m increases) when there is no branching dynamics. Similarly, the term $2\varepsilon\log(m_P m_Q)$ in (13) was not a part of the definition in the main text, but it is constant with given data. These changes are required to make the discrete cost consistent with the continuous cost appearing in the proof below where the entropy regularisation involves a Kullback–Leibler divergence. Without this term, the entropy term tends to $-\infty$ when $m_P, m_Q\to\infty$. In this section we make the notational distinction between the cost function variables (A,b) and the true system parameters $(\bar{A},\bar{b})$ that are assumed to have generated the observed data.

Assume the data is produced by a discrete-time system

$$x(k\Delta t)=(I+\Delta t\bar{A})x((k-1)\Delta t)+\Delta t\bar{b}+w_k \tag{14}$$

where $w_k\sim\mathcal{N}(0,\Delta t\varepsilon_0 I)$, $\varepsilon_0>0$, and $w_k\perp w_j$ if $j\neq k$, and the initial state is a sample from a normal distribution $x(0)\sim\mathcal{N}(m_0,P_0)$. Assume that each measured cell is an independent realisation of this process. Each cell measured at time $k\Delta t$ is then an independent sample from a normal distribution $\mathcal{N}(m_k,P_k)$ where m_k and P_k satisfy the moment equations

$$\begin{cases} m_k=(I+\Delta t\bar{A})m_{k-1}+\Delta t\bar{b}\\ P_k=(I+\Delta t\bar{A})P_{k-1}(I+\Delta t\bar{A}^\top)+\Delta t\varepsilon_0 I.\end{cases} \tag{15}$$

Here it is assumed that each gene has the same input noise intensity. General noise covariance $\varepsilon_0 R>0$ can be handled by using R^{-1} as a weight matrix in the Euclidean norm in (13).

Theorem 1. *Assume that the data consists of independent samples from a linear discrete-time system (14) and that at least three consecutive time points ($k=0,1,2,...$) have been measured with the following assumptions on the means and covariances of the measured distributions:*

(i) $P_0>0$ and $I+\Delta t\bar{A}$ is invertible;

(ii) The algebraic multiplicity of any eigenvalue of $P_0^{-1/2}P_1P_0^{-1/2}$ does not exceed two;

(iii) $P_0^{-1/2}(m_1-m_0)$ is not orthogonal to any eigenvector of $P_0^{-1/2}P_1P_0^{-1/2}$. In case an eigenvalue has multiplicity two, then $P_0^{-1/2}(m_1-m_0)$ is not orthogonal to the subspace spanned by the corresponding eigenvectors.

Assume further that the number of cells measured at each time point tends to infinity. Then the cost function (12) *converges in L^1 (in stochastic sense) to a continuous cost function whose unique global minimiser is the true system $(\bar{A}, \bar{b})$, provided that the noise intensity $\varepsilon = \Delta t \varepsilon_0$ is used as the entropy regularisation parameter.*

Assumptions (i)–(iii) are needed for unique identifiability of the model, and they are precisely the assumptions in [2, Corollary 2.1]. If they are violated, there may exist several systems that can produce the observed data (in the case of normally distributed data). In this case, no method can be guaranteed to find the true system.

In the method, the regularisation constants Λ_A and λ_b do not scale up with the number of cells measured, whereas the transport cost does. Therefore their effect vanishes at the infinite-data limit, and the theorem holds for the method as such. However, the noise intensity ε is never known in a real application and it is estimated from the data.

Proof. In the cost function (12), cells measured at time $k-1$ are propagated using a deterministic model, but they are compared to cells measured at time k that have evolved according to stochastic dynamics (14). This discrepancy is accounted for by the entropy regularisation, which corresponds exactly to the noise in the dynamics of the true data.

The proof consists of three parts. In the first part, it is established that as the number of cells tends to infinity, the discrete entropy-regularised transport cost defined for point clouds sampled from probability distributions converges to the continuous transport cost defined for the distributions. In the second part, we show that for given normal distribution $\mathcal{N}(m_a, P_a)$ where $P_a > \varepsilon I$, the normal distribution $\mathcal{N}(m_b, P_b)$ with $m_b = m_a$ and $P_b = P_a - \varepsilon I$ is the unique minimiser of the entropy-regularised transport cost between $\mathcal{N}(m_a, P_a)$ and $\mathcal{N}(m_b, P_b)$. In the third part, relying on our earlier identifiability result, it is shown that the true system $(\bar{A}, \bar{b})$ is the unique system producing minimal entropy-regularised transport cost.

Part 1. Let $\alpha_N = \{a_1, ..., a_N\}$ and $\beta_N = \{b_1, ..., b_N\}$, be collections of independent samples from two sub-Gaussian probability distributions, $a_j \sim \alpha$ and $b_j \sim \beta$. In [20, Theorem 2], it is shown that the discrete entropy-regularised optimal transport cost $W(\alpha_N, \beta_N)$ defined in (13) converges with rate $N^{-1/2}$ in L^1 as $N \to \infty$ to the continuous transport cost (using the notation of [24] which we also refer to for details on the continuous problem)[1]

$$\mathcal{L}^\varepsilon(\alpha, \beta) = \min_{\pi \in \mathcal{U}(\alpha, \beta)} \int_{\mathcal{X} \times \mathcal{Y}} \|x - y\|^2 \pi(x, y) dx\, dy + 2\varepsilon \mathrm{KL}(\pi | \alpha \otimes \beta)$$

where $\mathcal{U}(\alpha, \beta)$ is the set of feasible transport plans, that is, probability distributions in $\mathbb{R}^n \times \mathbb{R}^n$ satisfying the marginal constraints $\int \pi(x, y) dy = \alpha(x)$ and $\int \pi(x, y) dx = \beta(y)$. The stochastic L^1 convergence means $\mathbb{E}\big(|W(\alpha_N, \beta_N) - \mathcal{L}^\varepsilon(\alpha, \beta)|\big) \to 0$ as $N \to \infty$.

Part 2. The proof of part 2 relies on a closed form expression of $\mathcal{L}^\varepsilon(\alpha, \beta)$ in case α and β are normal distributions [14]:

$$\begin{aligned}\mathcal{L}^\varepsilon\big(\mathcal{N}(m_a, P_a), \mathcal{N}(m_b, P_b)\big) = \|m_a - m_b\|^2 + \mathrm{Tr}(P_a) + \mathrm{Tr}(P_b) - \mathrm{Tr}(D)\\ + n\varepsilon(1 - \log(2\varepsilon)) + \varepsilon \log(\det(D + \varepsilon I))\end{aligned}$$

where $D = \big(4P_a^{1/2} P_b P_a^{1/2} + \varepsilon^2 I\big)^{1/2}$. The task is to find (m_b, P_b) that minimise the transport cost for given (m_a, P_a) where $P_a > \varepsilon I$. Clearly, the minimising m_b is given by $m_b = m_a$. It is

[1] As we only discuss Gaussian distributions, we write the continuous OT problem in terms of density functions instead of general probability measures.

easier to find the minimum initially as a function of D rather than P_b. To perform this change of variables, the covariance P_b is expressed as a function of D:

$$P_b = \frac{1}{4} P_a^{-1/2}(D^2 - \varepsilon^2 I)P_a^{-1/2}.$$

The mapping between $D \geq \varepsilon I$ and $P_b \geq 0$ is bijective. Collecting the terms from the transport cost that depend on D yield the minimisation problem

$$\min_{D \geq \varepsilon I} \frac{1}{4}\mathrm{Tr}\big(P_a^{-1/2} D^2 P_a^{-1/2}\big) - \mathrm{Tr}(D) + \varepsilon \log(\det(D + \varepsilon I)). \tag{16}$$

The target function is differentiable with respect to D and the zeros of the derivative can be solved as follows (we refer to [23] for the derivative calculations):

$$\begin{aligned} &\frac{1}{2} P_a^{-1} D - I + \varepsilon(D + \varepsilon I)^{-1} = 0 \\ \Leftrightarrow\ & \frac{1}{2} P_a^{-1} D^2 + \frac{\varepsilon}{2} P_a^{-1} D - D = 0 \\ \Leftrightarrow\ & D = 2P_a - \varepsilon I \end{aligned}$$

where the last equivalence holds since D is invertible. The cost function tends to infinity when $\mathrm{Tr}(P_b) \to \infty$, and therefore the minimum must be obtained either at the boundary of the feasible set $\{D \geq \varepsilon I\}$, or at $D = 2P_a - \varepsilon I$ which would yield the desired result $P_b = P_a - \varepsilon I$.

It is next shown that there can not be even a local minimum on the boundary of the feasible set. A matrix on the boundary has the singular value decomposition $D = USU^\top$ where U is orthogonal, S is diagonal with $S_{j,j} \geq \varepsilon$, and $S_{i,i} = \varepsilon$ for some i. It will be shown that the derivative of the cost function with respect to $S_{i,i}$ is negative, meaning that there exists a matrix inside the feasible set that attains a lower cost value than the matrix on the boundary. It holds that $\mathrm{Tr}(D) = \sum_{j=1}^n S_{j,j}$ and so $\frac{d}{dS_{i,i}}\big(-\mathrm{Tr}(D)\big) = -1$. The last term in (16) can be written in terms of the decomposition by $\varepsilon \log(\det(D + \varepsilon I)) = \varepsilon \sum_{j=1}^n \log(S_{j,j} + \varepsilon)$ and so $\frac{d}{dS_{i,i}}\big(\varepsilon \log(\det(D + \varepsilon I))\big)\big|_{S_{i,i}=\varepsilon} = 1/2$. Finally, for the first term in (16), substituting $D^2 = US^2U^\top$, it holds that

$$\frac{d}{dS_{i,i}} \mathrm{Tr}\left(\frac{1}{4} P_a^{-1/2} U S^2 U^\top P_a^{-1/2}\right)\Bigg|_{S_{i,i}=\varepsilon} = \frac{\varepsilon}{2} U_i^\top P_a^{-1} U_i < \frac{1}{2}$$

where the inequality follows from $P_a > \varepsilon I$. Here U_i denotes the ith column of U. It follows that at any point on the boundary of the feasible set, the transport cost decreases when moving to the interior of the feasible set in such way that D moves to the direction $U_i U_i^\top$. Therefore $P_b = P_a - \varepsilon I$ must be the unique global minimum.

Part 3. Time point k consists of (infinite number of) samples from the normal distribution $\mathcal{N}(m_k, P_k)$ where m_k and P_k satisfy the moment equations (15). Based on part 2 of the proof, the normal distribution that minimises $\mathcal{L}^\varepsilon\big(\mathcal{N}(m_k, P_k), \mathcal{N}(m, P)\big)$ is given by $m = m_k = (I + \Delta t\bar{A})m_{k-1} + \Delta t\bar{b}$ and $P = P_k - \varepsilon I = (I + \Delta t\bar{A})P_{k-1}(I + \Delta t\bar{A}^\top)$. Note that by assumption (i), $P_k > \varepsilon I$ for $k \geq 1$.

In the method, samples measured at time point $k-1$ stored in matrix Y_{k-1} are propagated by the candidate model (A, b) by $(I + \Delta tA)Y_{k-1} + \Delta tb$. Since these are samples from a normal distribution $\mathcal{N}(m_{k-1}, P_{k-1})$, then the propagated points are samples from $\mathcal{N}\big((I + \Delta tA)m_{k-1} + \Delta tb, (I + \Delta tA)P_{k-1}(I + \Delta tA^\top)\big)$. If $(A, b) = (\bar{A}, \bar{b})$, this is exactly the distribution minimising the transport cost to the distribution of measurements of the next time point. Based on [2, Corollary 2.1], the true system is the only possible model to produce the minimising distribution.

□

Part 2 of the proof shows that the entropy regularisation deconvolutes the noise εI out of the observations, whose covariance is $(I+\Delta t\bar{A})P_{k-1}(I+\Delta t\bar{A}^{\top})+\varepsilon I$. In the case of normal distributions, deconvolution is a simple subtraction of the noise covariance. This idea of entropy regularisation as noise deconvolution holds more generally and it has been explored in [27].

The theorem is a consistency result stating that the true system matrices are the unique global minimiser of the cost function (12). However, there is no guarantee that the method will necessarily converge to the global minimum instead of a local minimum. The method is initialised from $(A, b) = (0, 0)$, whereby in the first iteration of the algorithm the optimal transport problem is solved directly between the measured time points without any propagation through a candidate model. If the transport mapping obtained in this initial step is poorly representing true cell propagation, then there is a risk that the method does not find the global minimum. The further apart the time points are from each other and the more of the developmental dynamics are not observed, the higher is the risk of this happening.

A recent preprint [11] proves a related theorem of identifiability for continuous-time systems. The relation between our assumptions and theirs is not immediately clear. In both theorems, the assumptions need to exclude rotational dynamics around a steady state that are not observable from distribution data that is "too symmetric".

Supplementary note 5: Data generation for the experiments of Sections 3.2–3.3

5.1 Linear dynamics

Data is simulated either using discrete-time dynamics

$$x(k\Delta T) = (I + \Delta T A)x((k-1)\Delta T) + \Delta T b + w_k$$

where $w_k \sim \mathcal{N}(0, \Delta T Q)$, and measurement times are exactly $k\Delta T$ with $k = 0, 1, ..., N$, or using continuous-time dynamics

$$\frac{d}{dt}x(t) = Ax(t) + b + w(t)$$

where w is a white noise process with covariance Q. The continuous-time system is simulated with Euler–Maruyama method using time step 0.01, which is well below the time scales of the measurements. The noise covariance used in data generation is $Q = qI$ where $q = \kappa^2 \text{mean}_j\big(-2A_{jj}x_{\text{ss},j}\big)$ where $-2A_{jj}x_{\text{ss},j}$ corresponds to the intensity of the Langevin noise for gene j at steady state x_{ss} for the nonlinear system (see the following section). We use $\kappa^2 = 0.02$ for noise intensity modulation. With this choice, the noise intensity for the linear and nonlinear models are comparable.

To generate single-cell data, each cell is independently drawn from an initial distribution, and then propagated through the stochastic model for the time specified by the measurement time, at which the value of the trajectory is stored.

The system A, b is given by

$$A = \begin{bmatrix} -1.5 & 0 & 0 & 0 & 0 & 0 & 0.4053 & 0 & 0 & 0.4053 \\ 0.2405 & -1.5 & -0.3269 & 0 & 0 & 0 & 0 & 0 & 0 & 0 \\ 0 & 0.4887 & -0.4 & 0 & 0 & 0 & 0 & 0 & 0 & 0 \\ 0 & 0 & 0.1509 & -1.5 & 0 & 0 & 0 & 0 & 0 & 0 \\ 0 & 0 & 0 & 0.6827 & -1.5 & 0 & 0 & 0 & 0 & 0 \\ 0 & 0 & 0.2658 & 0 & 0.2658 & -1.5 & 0 & 0 & 0 & 0 \\ 0 & 0 & 0 & 0 & 0 & 0.5555 & -2 & 0 & -0.4315 & 0 \\ 0 & 0 & 0 & 0 & 0 & 0 & 0.3080 & -0.8 & 0 & 0 \\ 0 & 0 & 0 & 0 & 0 & 0 & 0 & 0.7881 & -0.5 & 0 \\ 0 & 0 & 0 & 0 & 0 & 0 & 0 & 0 & 0.4675 & -1.2 \end{bmatrix}$$

$$b = \big[3.4750,\ 2.4679,\ 1.1554,\ 2.7959,\ 1.0879,\ 2.4127,\ 1.3674,\ 0.4087,\ 3.5551\big]^\top.$$

This system is based on the system used in [1]. However, to study the effect of nonlinearities in the system dynamics, a nonlinear system was first generated, which is described below. The linear system has been obtained by linearising the nonlinear system at the system's steady state. This way, the behaviours of the linear and nonlinear systems are highly similar. Time step $\Delta T = 0.4$ is used in the experiment and six time points are generated with 1000, 2000, 3000, 5000, 7500, and 10000 cells in each time point in the experiment in Section 3.2 and 600 cells in each time point in the experiments of Section 3.3. The initial states of each cell are drawn from a normal distribution $\mathcal{N}(m_0, P_0)$ where each entry of m_0 is drawn from a uniform distribution

$U(0, 2x_{\mathrm{ss},j})$. The initial state covariance is randomised by the following procedure:

$$\begin{aligned}
R_0 &= \mathrm{randn}(10, 10) && \text{(randomisation)}\\
R_1 &= R_0 R_0^\top && (\rightarrow \text{symmetric, pos. def.})\\
R_2 &= \mathrm{diag}(R_1)^{-1/2} R_1 \mathrm{diag}(R_1)^{-1/2} && \text{(normalisation)}\\
P_0 &= \mathrm{diag}(m_0)^{1/2} R_2 \mathrm{diag}(m_0)^{1/2} + 0.3I && \text{(scaling)}
\end{aligned}$$

where $\mathrm{randn}(m, n)$ produces an $m \times n$ matrix with entries independently drawn from $\mathcal{N}(0, 1)$.

5.2 Nonlinear dynamics

To demonstrate the effect of nonlinearity in the system dynamics, we created a nonlinear system by replacing all activations in the network with saturating Michaelis–Menten kinetics, where the saturation levels were chosen in a way that nonlinearity has an effect on the dynamics. Moreover, the two negative elements in the A-matrix above were replaced by proper inhibitions in the model.

The dynamics of each gene are governed by the Langevin equation ([10])

$$\frac{dx_j}{dt} = -a_j x_j + f_j(x) + \kappa\big(\sqrt{a_j x_j}\, w_j + \sqrt{f_j(x)}\, v_j\big) \tag{17}$$

where w_j and v_j are independent white noise processes corresponding to uncertainty in RNA degradation and expression processes, respectively. The noise scaling coefficient κ is related to the molecule numbers within a cell. The higher the molecule numbers are, the smaller is the effect of noise. If the state vector directly corresponds to actual molecule numbers, then $\kappa = 1$ should be used. We, however, use it as a tuning parameter and set $\kappa = \sqrt{0.02}$. The functions f_j are gene expression rates and are therefore always non-negative. Without the noise, any simulation that is initiated from non-negative values in $x(0)$ will remain non-negative. Since the Langevin-approximation of the chemical master equation may violate the non-negativity assumption, after every time step in the numerical simulation, possible negative values are set to zero. The functions f_j for $j = 1, ..., 10$ are as follows:

$$\begin{aligned}
f_j(x) &= \tfrac{v_j + k_j x_{j-1}}{1 + c_j x_{j-1}}, && \text{for } j = 3, 4, 5, 8, 9, 10,\\
f_1(x) &= \tfrac{v_1 + k_1(x_7 + x_{10})}{1 + c_1(x_7 + x_{10})}, && f_2(x) = \tfrac{v_2 + k_2 x_1}{(1 + c_2 x_1)(1 + d_2 x_3)},\\
f_6(x) &= \tfrac{v_6 + k_6(x_3 + x_5)}{1 + c_6(x_3 + x_5)}, && f_7(x) = \tfrac{v_7 + k_7 x_6}{(1 + c_7 x_6)(1 + d_7 x_9)}.
\end{aligned}$$

This nonlinear system has precisely the same regulatory interactions as the linear system above. That is, six genes (3,4,5,8,9,10) are activated by one gene ($x_{j-1} \rightarrow x_j$). Genes 1 and 6 are activated by two genes ($x_7, x_{10} \rightarrow x_1$ and $x_3, x_5 \rightarrow x_6$). Genes 2 and 7 have one activation and one inhibition ($x_3 \dashv x_2$, $x_1 \rightarrow x_2$ and $x_9 \dashv x_7$, $x_6 \rightarrow x_7$). The parameter values of the system are shown in the adjacent table.

To ensure that the linear and nonlinear dynamics would be as close to each other as possible (that is, nonlinearity being the only difference), same noise process realisations were used in the simulations. Since the nonlinear system (17) has two noise processes w_j and v_j per gene, for the linear dynamics, $(w_j + v_j)/\sqrt{2}$ was used as the noise process. Then the noise intensities of the linear and nonlinear systems are comparable with each other (note that $a_j x_{\mathrm{ss},j} = f_j(x_{\mathrm{ss}})$).

5.3 Protein dynamics

Typically the RNA-molecules do not act as transcription factors directly, but are first translated into proteins. The protein levels, however, are typically not measured, and computational methods directly fit models between RNA concentrations. To test the effect of this omission, we modified the nonlinear system by introducing a variable mimicking protein concentrations whose dynamics are governed by

$$\frac{d}{dt}p(t) = L(x(t) - p(t)), \qquad p(0) = x(0)$$

where L is a diagonal matrix with strictly positive diagonal entries, that are drawn from $U(0.5, 1)$ separately for each replicate. In the RNA dynamics (17), p replaces x as the variable of the functions f_j. No noise is considered in the protein dynamics, since we only wished to study the effect of the delay introduced by protein dynamics while ignoring the effect of additional noise. The initial state $p(0) = x(0)$ is chosen following an assumption that the population is in a steady state before a perturbation is introduced, and the protein levels have converged to correspond to this steady state (note that if $x(t) = x_c$ is constant, then $p(t) \to x_c$ as $t \to \infty$).

With the protein concentrations included, the system's dimension is 20, but the method is applied on the 10-dimensional data of RNA concentrations, and the protein concentrations are ignored.

5.4 Mutation and perturbation model

The method can be used to find targets of external perturbations (for example, a drug) or pathways affected by a mutation. To generate data for validating the approach, the nonlinear model was slightly modified. In the original model, no gene regulates more than two other genes. To have a more meaningful validation experiment for mutation effects, gene 9 (which already activates gene 10 and inhibits gene 7) is given a third regulation, namely an activation of gene 4. In addition, the three regulatory effects of gene 9 are modulated by coefficients $s_i \in [0, 1]$, for $i = 1, 2, 3$, simulating a partial loss of function. That is, functions f_4, f_7, and f_{10} in the model are replaced by

$$f_4(x) = \tfrac{v_4 + k_4(x_3 + s_1 x_9)}{1 + c_4(x_3 + s_1 x_9)}, \quad f_7(x) = \tfrac{v_7 + k_7 x_6}{(1 + c_7 x_6)(1 + d_7 s_2 x_9)}, \quad f_{10}(x) = \tfrac{v_{10} + k_{10} s_3 x_9}{1 + c_{10} s_3 x_9}.$$

This model was then run always first with $s_1 = s_2 = s_3 = 1$ to simulate a system without any mutation. Then, another experiment was simulated with setting one or two of the three

Table: Parameters of the nonlinear model.

Gene (j)	a_j	k_j	v_j	c_j	d_j
1	1.5	2.0991	0.4524	0.2015	
2	1.5	3.6469	0.5124	0.2318	0.6667
3	0.4	2.5882	0.4377	0.8641	
4	1.5	3.9127	0.0479	0.9068	
5	1.5	1.5617	0.3310	0.2069	
6	1.6	3.6369	0.1265	0.4267	
7	2.0	2.2910	0.3932	0.0411	0.4545
8	0.8	3.7228	0.4238	1.5239	
9	0.5	1.0370	0.1570	0.0628	
10	1.2	3.2482	0.3040	0.3638	

coefficients to either 0.5 or 0.75. The initial distribution for the control and mutation datasets were the same. Ten replicates for each gene combination were simulated, that is, altogether 30 replicates for each of the four cases (50% loss of function for one regulation, 50% loss for two regulations, 25% loss for one regulation, and 25% loss for two regulations).

To simulate the effect of perturbations, the basal transcription rates v_j were modulated by coefficients $r_j \geq 1$. As with the mutation dataset, one experiment was simulated without the perturbation ($r_j = 1$ for all j), and then another experiment with a perturbation. Different number (one, two, or three) of genes were randomly chosen as perturbation targets, and the coefficients r_j were increased to either 1.3 or 1.6 for the affected genes, corresponding to 30% or 60% increase in the basal transcription rate. For each of the six combinations of number of affected genes and perturbation strengths, 20 replicates were simulated with different perturbation targets.

References

[1] A. Aalto and J. Gonçalves. Linear system identification from ensemble snapshot observations. In *2019 IEEE 58th Conference on Decision and Control (CDC)*, pages 7554–7559, 2019. doi:10.1109/CDC40024.2019.9029334.

[2] A. Aalto, F. Lamoline, and J. Gonçalves. Linear system identifiability from single-cell data. *Systems & Control Letters*, 165:105287, 2022. doi:10.1016/j.sysconle.2022.105287.

[3] Y. Benjamini and Y. Hochberg. Controlling the false discovery rate: Practical and powerful approach to multiple testing. *Journal of the Royal Statistical Society B*, 57(1):289–300, 1995. doi:10.1111/j.2517-6161.1995.tb02031.x.

[4] Y. Chen and J. Karlsson. State tracking of linear ensembles via optimal mass transport. *IEEE Control Systems Letters*, 2(2):260–265, 2018. doi:10.1109/LCSYS.2018.2827001.

[5] Y. Chen, T. T. Georgiou, and M. Pavon. Optimal transport over a linear dynamical system. *IEEE Transactions on Automatic Control*, 62(5): 2137–2152, 2016. doi:10.1109/TAC.2016.2602103.

[6] Y. Chen, T. T. Georgiou, and M. Pavon. On the relation between optimal transport and Schrödinger bridges: A stochastic control viewpoint. *Journal of Optimization Theory and Applications*, 169(2):671–691, 2016. doi:10.1007/s10957-015-0803-z.

[7] M. A. Coomer, L. Ham, and M. P. Stumpf. Noise distorts the epigenetic landscape and shapes cell-fate decisions. *Cell Systems*, 13(1):83–102.e6, 2022. doi:10.1016/j.cels.2021.09.002.

[8] M. Cuturi. Sinkhorn distances: Lightspeed computation of optimal transport. *Advances in Neural Information Processing Systems*, 26:2292–2300, 2013.

[9] J. Dusonchet, H. Li, M. Guillily, M. Liu, K. Stafa, C. Derada Troletti, J. Y. Boon, S. Saha, L. Glauser, A. Mamais, et al. A Parkinson's disease gene regulatory network identifies the signaling protein RGS2 as a modulator of LRRK2 activity and neuronal toxicity. *Human Molecular Genetics*, 23(18):4887–4905, 2014. doi:10.1093/hmg/ddu202.

[10] D. Gillespie. The chemical Langevin equation. *Journal of Chemical Physics*, 113:297–306, 2000. doi:10.1063/1.481811.

[11] V. Guan, J. Janssen, H. Rahmani, A. Warren, S. Zhang, E. Robeva, and G. Schiebinger. Identifying drift, diffusion, and causal structure from temporal snapshots. *arXiv*, page 2410.22729v2, 2024. doi:10.48550/arXiv.2410.22729.

[12] I. Haasler, J. Karlsson, and A. Ringh. Control and estimation of ensembles via structured optimal transport. *IEEE Control Systems Magazine*, 41(4):50–69, 2021. doi:10.1109/MCS.2021.3076540.

[13] J. Hasenauer, S. Waldherr, M. Doszczak, P. Scheurich, N. Radde, and F. Allgöwer. Analysis of heterogeneous cell populations: A density-based modeling and identification framework. *Journal of Process Control*, 21(10):1417–1425, 2011. doi:10.1016/j.jprocont.2011.06.020.

[14] H. Janati, B. Muzellec, G. Peyré, and M. Cuturi. Entropic optimal transport between unbalanced Gaussian measures has a closed form. *Advances in Neural Information Processing Systems*, 33:10468–10479, 2020.

[15] M. Kanehisa, Y. Sato, M. Kawashima, M. Furumichi, and M. Tanabe. KEGG as a reference resource for gene and protein annotation. *Nucleic Acids Research*, 44:D457–D462, 2016. doi:10.1093/nar/gkv1070.

[16] L. Kantorovich. On the translocation of masses. *Doklady Akademii Nauk SSSR*, 37(7–8):227–229, 1942.

[17] L. Kolberg, U. Raudvere, I. Kuzmin, P. Adler, J. Vilo, and H. Peterson. g:Profiler-interoperable web service for functional enrichment analysis and gene identifier mapping (2023 update). *Nucleic Acids Research*, 51:W207–W212, 2023. doi:10.1093/nar/gkad347.

[18] G. La Manno, R. Soldatov, A. Zeisel, E. Braun, H. Hochgerner, V. Petukhov, K. Lidschreiber, M. E. Kastriti, P. Lönnerberg, A. Furlan, J. Fan, L. E. Borm, Z. Liu, D. van Bruggen, J. Guo, X. He, R. Barker, E. Sundström, G. Castelo-Branco, P. Cramer, I. Adameyko, S. Linnarsson, and P. V. Kharchenko. RNA velocity of single cells. *Nature*, 560:494–498, 2018. doi:10.1038/s41586-018-0414-6.

[19] C. Léonard. A survey of the Schrodinger problem and some of its connections with optimal transport. *Discrete and Continuous Dynamical Systems*, 34(4):1533–1574, 2014. doi:10.3934/dcds.2014.34.1533.

[20] G. Mena and J. Niles-Weed. Statistical bounds for entropic optimal transport: sample complexity and the central limit theorem. *Advances in Neural Information Processing Systems*, 32, 2019.

[21] G. Novak, D. Kyriakis, K. Grzyb, M. Bernini, S. Rodius, G. Dittmar, S. Finkbeiner, and A. Skupin. Single-cell transcriptomics of human iPSC differentiation dynamics reveal a core molecular network of Parkinson's disease. *Communications Biology*, 5:49, 2022. doi:10.1038/s42003-021-02973-7.

[22] B. Øksendal. *Stochastic Differential Equations: An Introduction with Applications*. Universitext. Springer Berlin Heidelberg, 2010. ISBN 9783642143946.

[23] K. B. Petersen and M. S. Pedersen. The matrix cookbook, 2012. URL `http://www2.compute.dtu.dk/pubdb/pubs/3274-full.html`. Version 20121115.

[24] G. Peyré and M. Cuturi. Computational optimal transport. *Foundations and Trends in Machine Learning*, 11(5-6):355–607, 2019. doi:10.1561/2200000073.

[25] A. Pratapa, A. Jalihal, J. Law, A. Bharadwaj, and T. M. Murali. Benchmarking algorithms for gene regulatory network inference from single-cell transcriptomic data [Data set]. Zenodo, 2020. doi:10.5281/zenodo.3701939.

[26] A. Pratapa, A. P. Jalihal, J. N. Law, A. Bharadwaj, and T. M. Murali. Benchmarking algorithms for gene regulatory network inference from single-cell transcriptomic data. *Nature Methods*, 17(2):147–154, 2020. doi:10.1038/s41592-019-0690-6.

[27] P. Rigollet and J. Weed. Entropic optimal transport is maximum-likelihood deconvolution. *Comptes Rendus. Mathématique*, 356(11–12):1228–1235, 2018. doi:10.1016/j.crma.2018.10.010.

[28] E. Schrödinger. *Über die Umkehrung der Naturgesetze*. Sitzungsberichte der Preussischen Akademie der Wissenschaften. Physikalisch-mathematische Klasse. Verlag der Akademie der Wissenschaften in Kommission bei Walter De Gruyter u. Company, 1931.

[29] J. Walter, S. Bolognin, S. K. Poovathingal, S. Magni, D. Gérard, P. M. Antony, S. L. Nickels, L. Salamanca, E. Berger, L. M. Smits, K. Grzyb, R. Perfeito, F. Hoel, X. Qing, J. Ohnmacht, M. Bertacchi, J. Jarazo, T. Ignac, A. S. Monzel, L. Gonzalez-Cano, R. Krüger, T. Sauter, M. Studer, L. P. de Almeida, K. J. Tronstad, L. Sinkkonen, A. Skupin, and J. C. Schwamborn. The Parkinson's-disease-associated mutation LRRK2-G2019S alters dopaminergic differentiation dynamics via NR2F1. *Cell Reports*, 37(3):109864, 2021. doi:10.1016/j.celrep.2021.109864.

[30] T. Zhang. Adaptive forward-backward greedy algorithm for learning sparse representations. *IEEE Transactions on Information Theory*, 57(7): 4689–4708, 2011. doi:10.1109/TIT.2011.2146690.

Supplementary table 1: Complete EPR results on the (log-transformed) BEELINE RNA-Seq data. The columns "TFs + 500 genes" and "TFs + 1000 genes" are comparable to [26, Fig. 5] and columns "500 genes" and "1000 genes" are comparable to [26, Suppl. fig. 8].

Ground truth	Data	TFs+ 500 genes	TFs+ 1000 genes	500 genes	1000 genes
STRING	mHSC-E	4.574	4.537	3.262	3.635
	mHSC-L	4.738	5.000	4.925	5.000
	mHSC-GM	6.119	6.409	4.912	5.790
	mESC	2.816	2.732	2.301	2.535
	mDC	1.459	1.629	1.283	1.160
Nonspecific ChIP-Seq	mHSC-E	3.159	3.039	1.679	2.429
	mHSC-L	2.054	2.182	1.831	2.182
	mHSC-GM	4.002	3.307	3.259	3.699
	mESC	2.443	2.936	1.730	2.076
	mDC	2.204	2.323	0.956	1.742
Celltype-specific ChIP-Seq	mHSC-E	1.005	1.002	1.009	1.001
	mHSC-L	1.023	1.010	1.025	1.010
	mHSC-GM	1.001	1.008	0.983	0.987
	mESC	1.033	1.012	1.031	1.043
	mDC	0.836	0.877	0.938	1.043
lof/gof	mESC	1.263	1.171	1.246	1.185
STRING	hESC	2.973	3.133	2.868	2.850
	hHep	2.048	1.921	1.763	2.062
Nonspecific ChIP-Seq	hESC	1.600	1.390	1.380	0.505
	hHep	1.337	1.240	1.826	1.827
Celltype-specific ChIP-Seq	hESC	1.082	1.125	1.070	0.937
	hHep	1.010	1.007	0.999	1.017

Supplementary table 2: Results of pathway enrichment analysis for the *LRRK2* dataset. The table shows statistically significant results (rank 1–3 where adjusted p-value < 0.05) as well as other terms with p-value < 0.05, KS > 0.4, and #genes > 1 to highlight some terms whose statistical significance is thwarted by the small term size. Here KS is the Kolmogorov–Smirnov statistic, which is then multiplied by $(\#\text{genes})^{1/2}$ to get the adjusted KS statistic. Adjusted p-values are obtained by multiplying the p-value by the number of pathways tested (291), and then dividing by the rank of each pathway. The column "top200" indicates the percentage of genes of the term that are on top-200 on the list given by GRIT.

Rank	KEGG term name	p_value	p_adj	KS_adj	KS	#genes	top200
1	Oxidative phosphorylation	9.541e-05	0.02776	2.097	0.3597	34	38.2
2	Thermogenesis	0.0004022	0.04919	1.936	0.3023	41	31.7
3	Diabetic cardiomyopathy	0.0005071	0.04919	1.903	0.3172	36	33.3
...							
6	Lysosome	0.00341	0.1654	1.56	0.637	6	50
7	IL-17 signaling pathway	0.005018	0.1727	1.468	0.734	4	75
11	Efferocytosis	0.01036	0.2741	1.354	0.782	3	66.7
12	Viral myocarditis	0.01266	0.2927	1.407	0.445	10	40
16	Autoimmune thyroid disease	0.0225	0.3637	1.202	0.85	2	100
17	Allograft rejection	0.0225	0.3637	1.202	0.85	2	100
18	Graft-versus-host disease	0.0225	0.3637	1.202	0.85	2	100
20	Type I diabetes mellitus	0.0411	0.532	1.166	0.583	4	75
22	Ferroptosis	0.04385	0.532	1.124	0.649	3	66.7
24	Gastric acid secretion	0.04609	0.532	1.162	0.4743	6	50

Supplementary table 3: Results of pathway enrichment analysis for the *PINK1* dataset. The table shows statistically significant results (rank 1–14 where adjusted p-value < 0.05) as well as other terms with p-value < 0.05, KS > 0.4, and #genes > 1 to highlight some terms whose statistical significance is thwarted by the small term size. Here KS is the Kolmogorov–Smirnov statistic, which is then multiplied by $(\#genes)^{1/2}$ to get the adjusted KS statistic. Adjusted p-values are obtained by multiplying the p-value by the number of pathways tested (316), and then dividing by the rank of each pathway. The column "top200" indicates the percentage of genes of the term that are on top-200 on the list given by GRIT.

Rank	KEGG term name	p_value	p_adj	KS_adj	KS	#genes	top200
1	Adrenergic signaling in cardiomyocytes	0.0001728	0.03229	1.999	0.4712	18	38.9
2	Vascular smooth muscle contraction	0.0002044	0.03229	1.95	0.5629	12	41.7
3	Motor proteins	0.0003829	0.03612	1.933	0.3471	31	41.9
4	Melanogenesis	0.0006452	0.03612	1.814	0.547	11	36.4
5	Dopaminergic synapse	0.0007539	0.03612	1.802	0.5203	12	41.7
6	Tuberculosis	0.0008241	0.03612	1.743	0.6589	7	28.6
7	Oocyte meiosis	0.0008907	0.03612	1.811	0.4049	20	30
8	Renin secretion	0.0009145	0.03612	1.686	0.7542	5	60
9	Long-term potentiation	0.00111	0.03897	1.71	0.6462	7	42.9
10	Amphetamine addiction	0.001257	0.03973	1.727	0.5462	10	40
11	Protein processing in endoplasmic reticulum	0.00143	0.04109	1.767	0.3076	33	27.3
12	cGMP-PKG signaling pathway	0.001569	0.04132	1.728	0.432	16	18.8
13	Lipid and atherosclerosis	0.001701	0.04134	1.725	0.3957	19	36.8
14	Oxytocin signaling pathway	0.002064	0.04659	1.694	0.4109	17	35.3
...							
15	Aldosterone synthesis and secretion	0.003324	0.07003	1.596	0.5319	9	55.6
16	Glucagon signaling pathway	0.004859	0.09597	1.55	0.4902	10	50
18	Calcium signaling pathway	0.006254	0.1098	1.527	0.4234	13	38.5
19	Inflammatory mediator regulation of TRP channels	0.007547	0.1199	1.474	0.5212	8	50
20	Prostate cancer	0.008085	0.1199	1.481	0.4465	11	36.4
22	Circadian entrainment	0.008411	0.1199	1.439	0.5875	6	66.7
23	Platelet activation	0.008728	0.1199	1.476	0.4095	13	23.1
24	GnRH signaling pathway	0.01046	0.1331	1.426	0.5042	8	50
25	Glycine, serine and threonine metabolism	0.01053	0.1331	1.269	0.8974	2	100
27	Alcoholism	0.01235	0.1445	1.411	0.4462	10	30
28	Long-term depression	0.01359	0.1534	1.249	0.8834	2	100
33	Progesterone-mediated oocyte maturation	0.01757	0.1683	1.354	0.4282	10	30
34	Insulin secretion	0.01825	0.1696	1.331	0.5031	7	42.9
37	Phosphatidylinositol signaling system	0.02824	0.2366	1.239	0.5542	5	40
38	Legionellosis	0.02845	0.2366	1.25	0.5102	6	33.3
39	Salivary secretion	0.03144	0.2548	1.239	0.4684	7	57.1
43	IL-17 signaling pathway	0.03729	0.2741	1.183	0.5914	4	50
44	Olfactory transduction	0.04015	0.2883	1.188	0.4849	6	33.3
46	Viral myocarditis	0.0423	0.2906	1.19	0.4209	8	50
47	Parathyroid hormone synthesis, secretion and action	0.04393	0.2954	1.178	0.4454	7	42.9

Supplementary table 4: Median computation times for the hHEP, hESC, and mHSC-E cases in the BEELINE RNA-seq benchmark (following the generation of Fig. 6 in the BEELINE benchmark). Computation times for other methods are from the BEELINE article. GRIT was applied using parallel computing with 12 processors (Dell workstation, 2.5 GHz Intel Xeon E5-2680 v3). The actual completion time is multiplied by 12 to get (upper bounds for) processing times shown in the table. Note that different methods have been run with different equipment, and therefore the table should be considered as indicative.

n	500	1000
GENIE3	1 h	3 h
GRNBOOST2	10 min	30 min
SCODE	5 min	5 min
GRISLI	1 h	3 h
GRIT	31 min	2 h

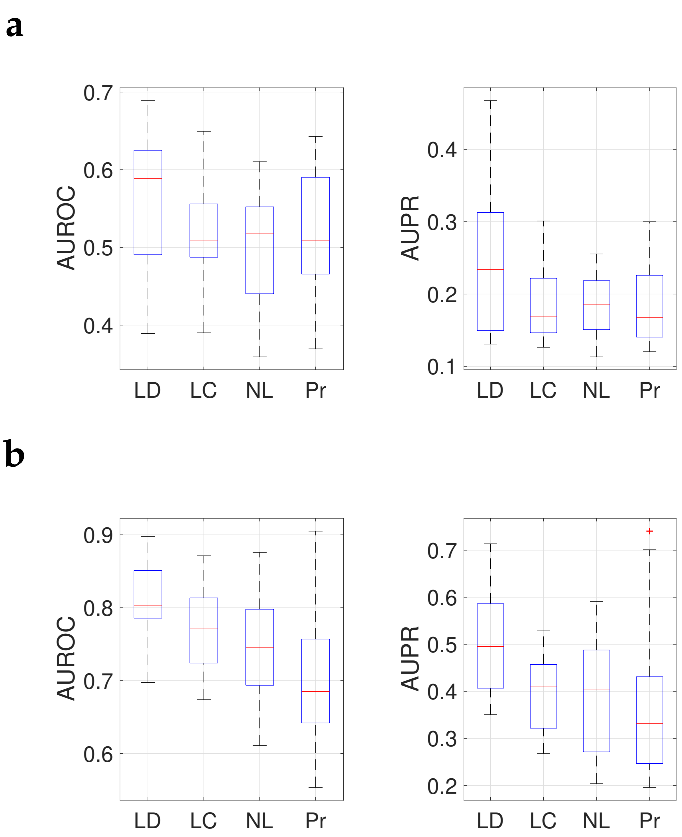

Supplementary figure 4: a: Results on the experiment of Section 3.3 by applying GRIT on bulk data, where each cell's expression vector has been replaced by the mean of the corresponding time point. **b:** Results on the experiment of Section 3.3 calculated from the absolute values of the A-matrix entries. These plots are directly comparable with Figure 2b in the main text.

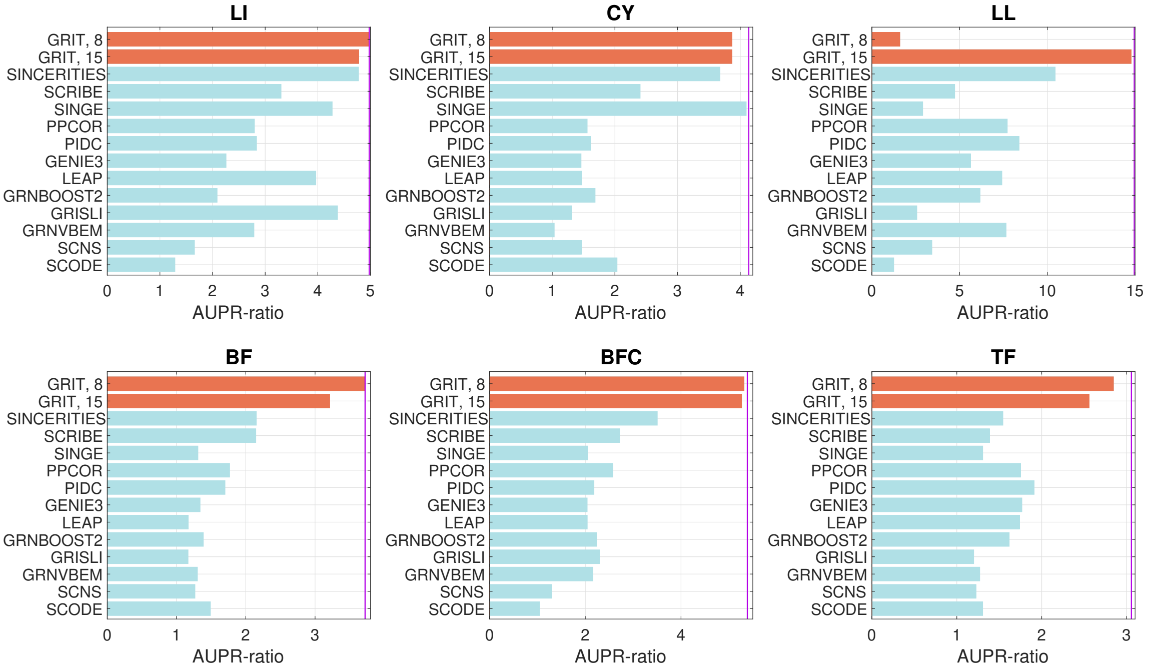

Supplementary figure 5: Detailed results of the BEELINE synthetic dataset. Bars show the median AUPR-ratios for cases with 2000 or 5000 cells (corresponding to [26, Fig. 2]). Results for the other methods are taken from the source file for [26, Fig. 2]. The purple line indicates the score for perfect reconstruction (the inverse of $\mathbf{E}$(AUPR) for random networks).

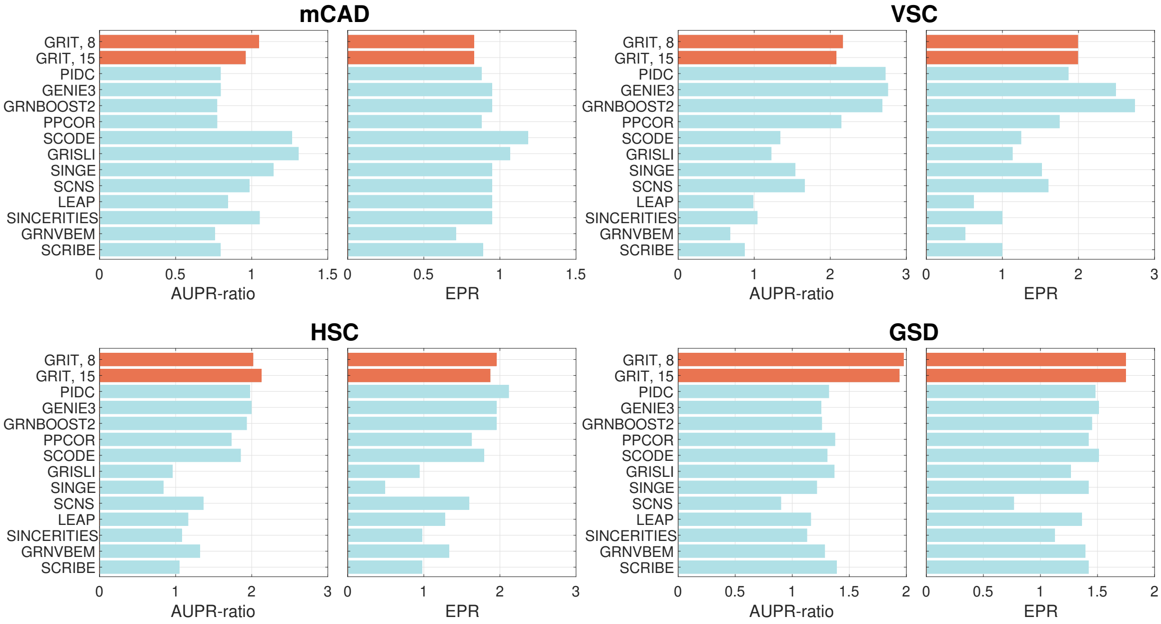

Supplementary figure 6: Detailed results of the BEELINE curated dataset. Bars show the median AUPR-ratios and EPRs for cases without dropouts (corresponding to [26, Fig. 4]). Results for the other methods are taken from the source file for [26, Fig. 4].

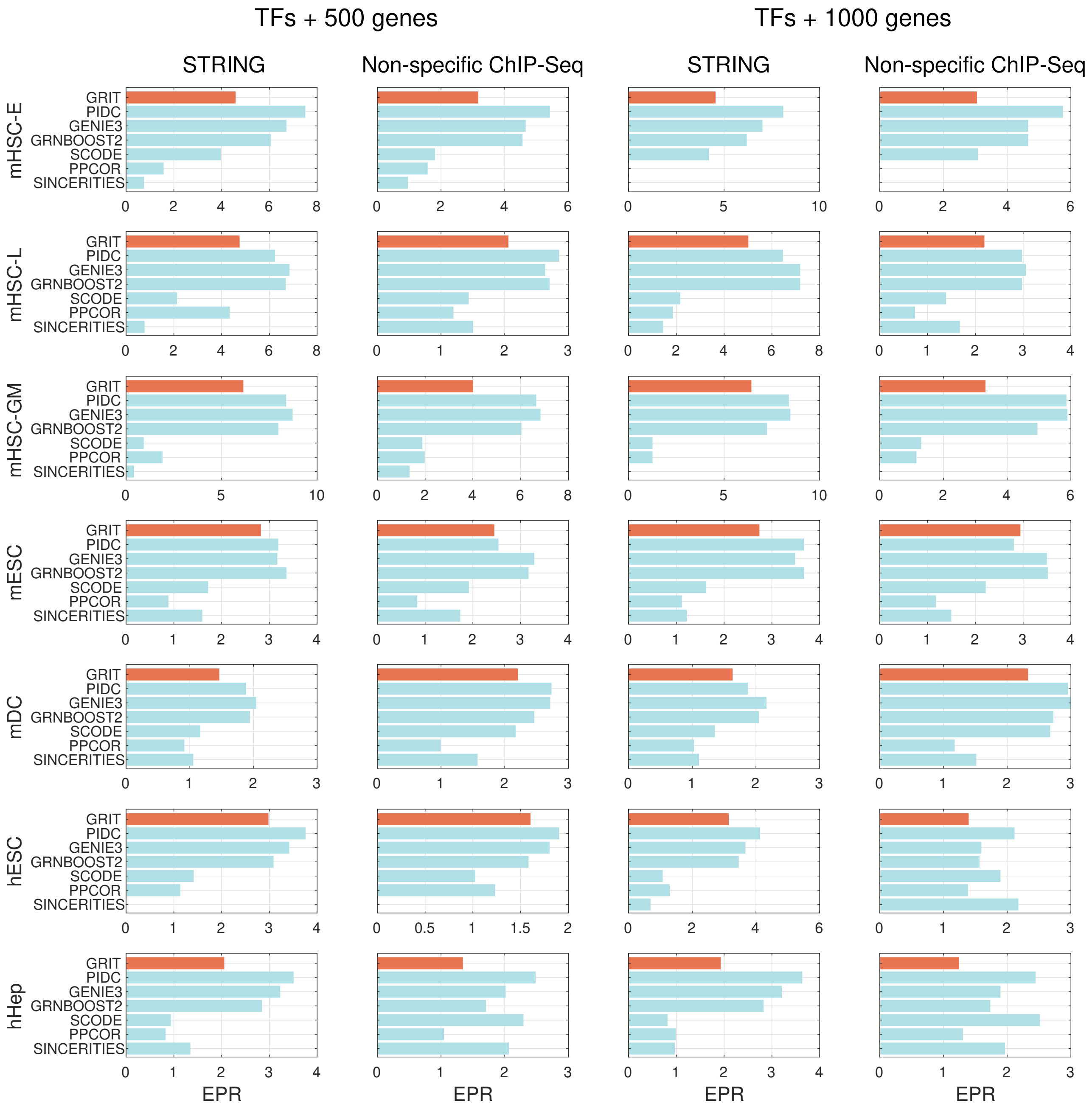

Supplementary figure 7: Detailed results of the BEELINE RNA-Seq datasets with the STRING and Non-specific ChIP-Seq ground truth networks (that seem to be the best ones for comparison). Complete results corresponding to [26, Fig. 5 and Suppl. Fig. 8] are in Supplementary table 2. Results for the other methods are taken from the source file for [26, Fig. 5].

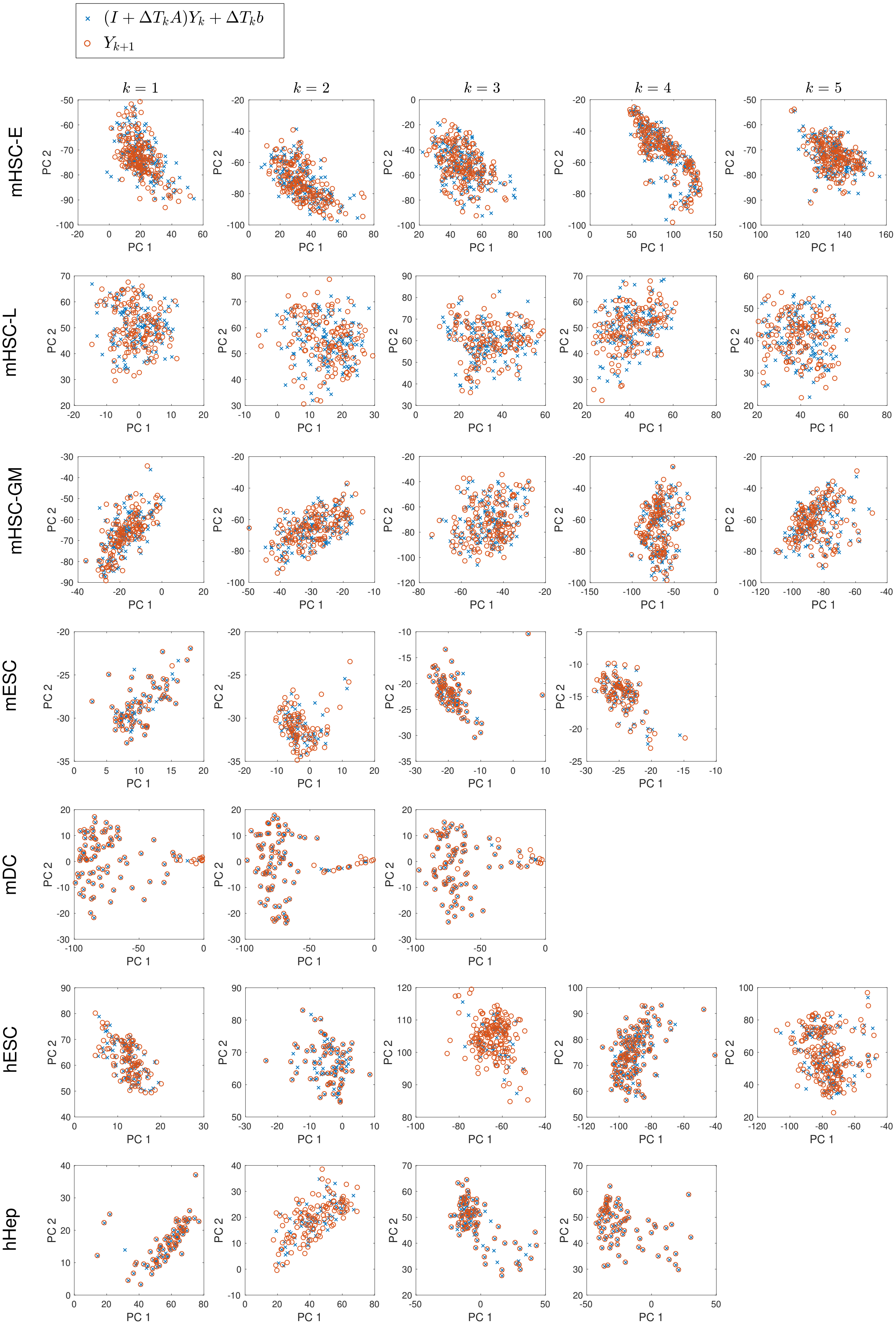

Supplementary figure 8: Propagation plots showing the propagated cells on time point k and the target cells on time point $k+1$ in the case with TFs and 500 most highly varying genes. Some overfitting can be observed in cases with low cell numbers. However, increased regularisation did not yield an improvement in performance (see Supplementary figure 11).

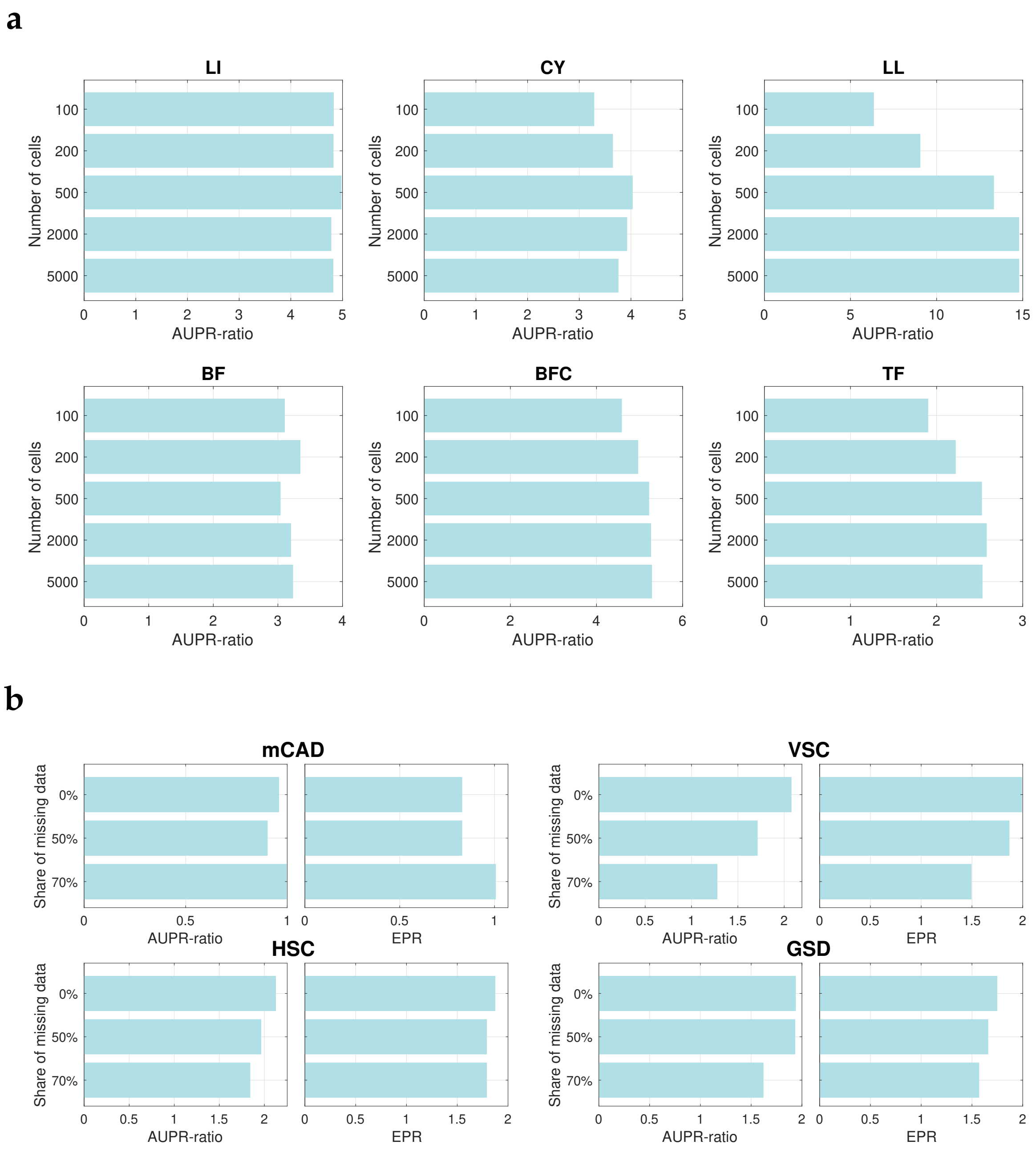

Supplementary figure 9: a: Effect of number of cells on the inference results of GRIT with 15 time points in the BEELINE synthetic dataset. **b:** Effect of dropout rate on the inference results of GRIT with 15 time points in the BEELINE curated dataset. The results are included for transparency, but it should be noted that the dropouts in BEELINE are generated by randomly selecting entries in the data matrices to be replaced by zero. This is not a realistic dropout model.

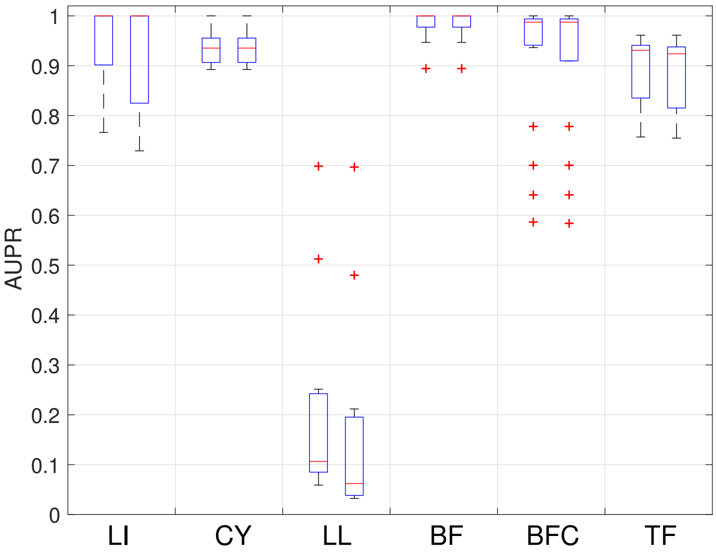

Supplementary figure 10: Signed predictions were evaluated on the BEELINE synthetic case (with data split to 8 time points). The AUPR performance metrics were calculated first for the non-signed predictions. Then, entries in the signed output matrix with wrong sign were zeroed and the performance metrics were calculated for these matrices. With this procedure, the AUPRs for the non-signed predictions are always higher than for the signed predictions. The box plots for each case show the AUPRs for non-signed (left) and signed predictions (right) including cases with 2000 or 5000 cells.

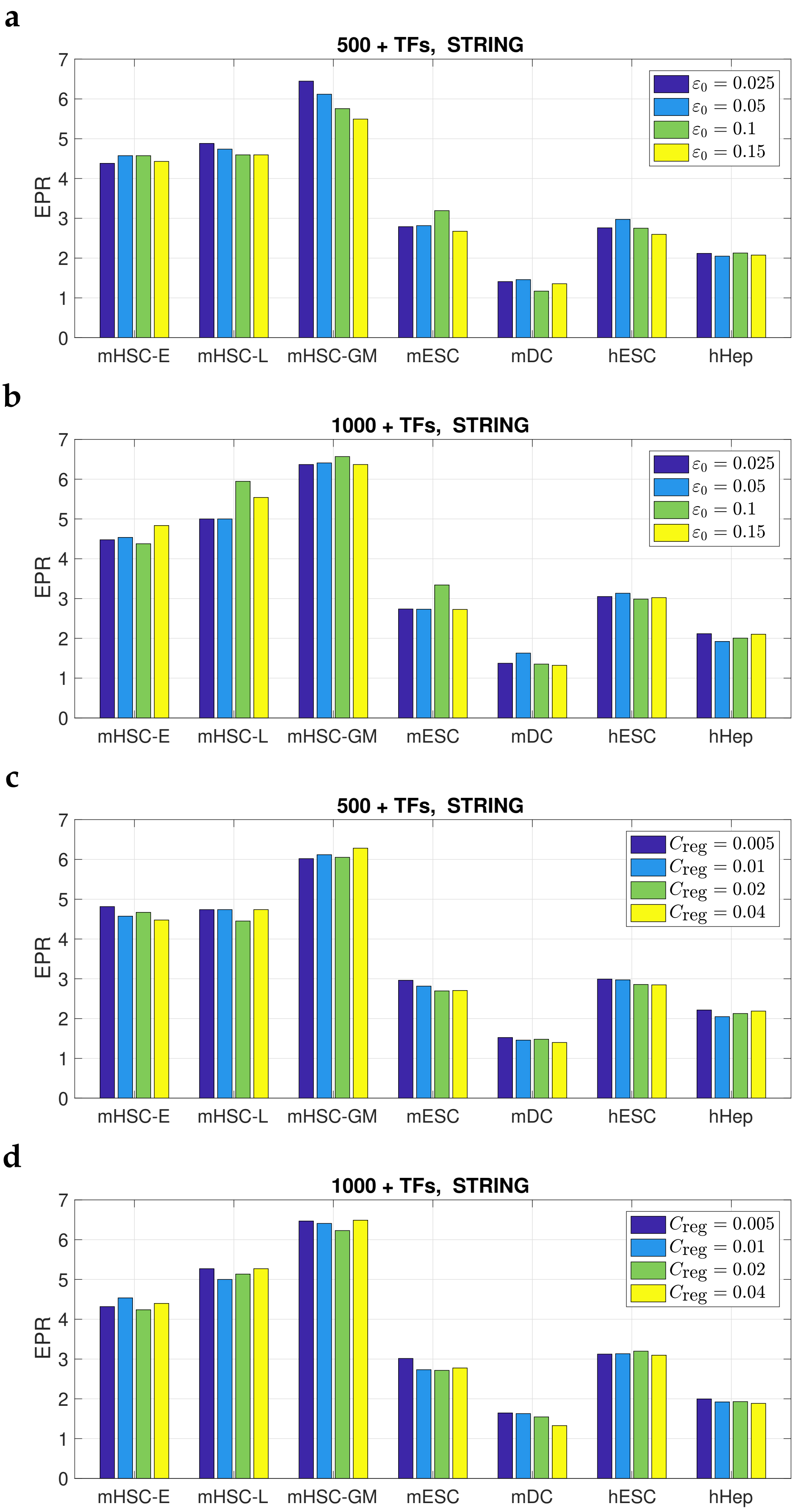

Supplementary figure 11: Effect of the entropy regularisation scaling coefficient ε_0 (**a**,**b**) and the regression regularisation coefficient C_{reg} (**c**,**d**) on the inference results (EPR for STRING ground truth) for the case with TFs and 500 most highly varying genes (**a**,**c**) and TFs and 1000 most highly varying genes (**b**,**d**).

a

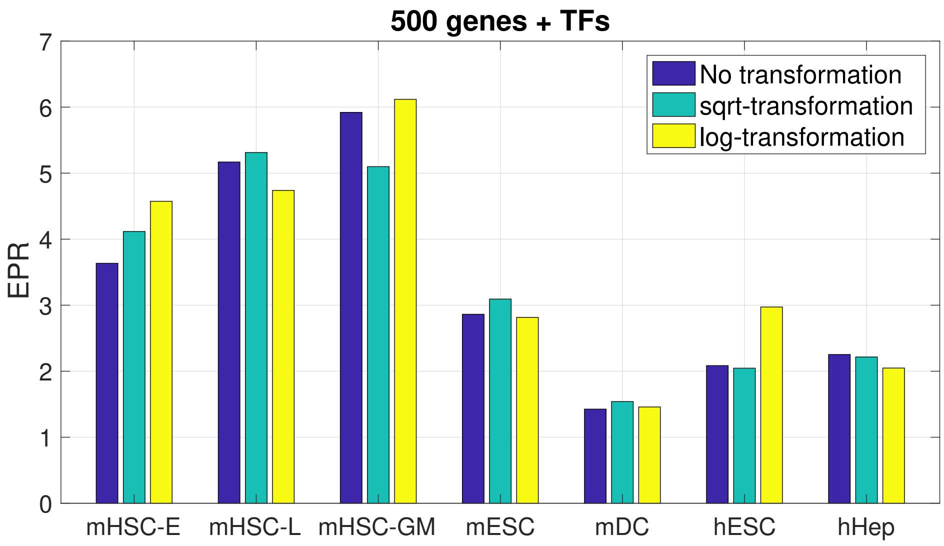

b

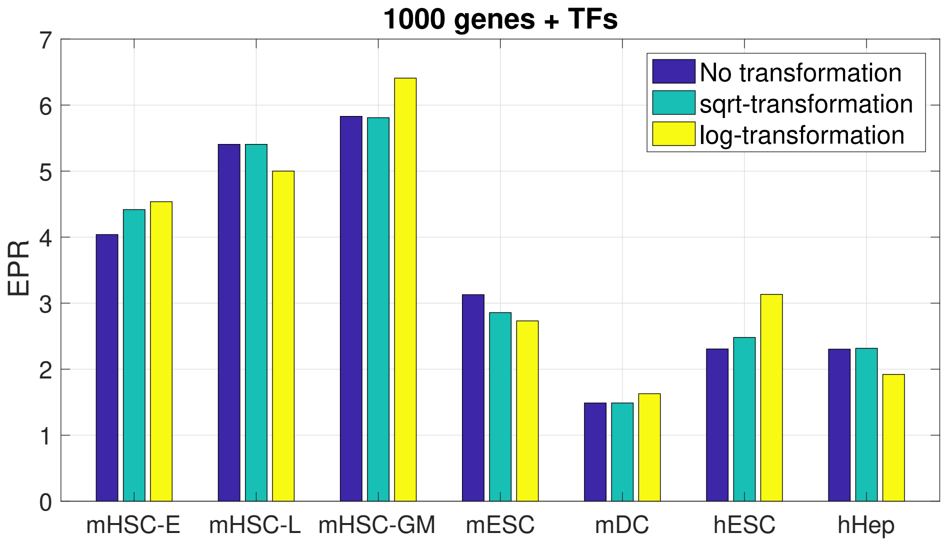

Supplementary figure 12: Effect of different transformations on the data on the inference results (EPR for STRING ground truth) for the case with TFs and 500 most highly varying genes (**a**) and TFs and 1000 most highly varying genes (**b**). The data as it is provided in BEELINE has been transformed by $y = \log_2(x+1)$. The case with no transformation is done by cancelling the log-transformation by calculating $x = 2^y - 1$ for each entry y in the data matrix.

a

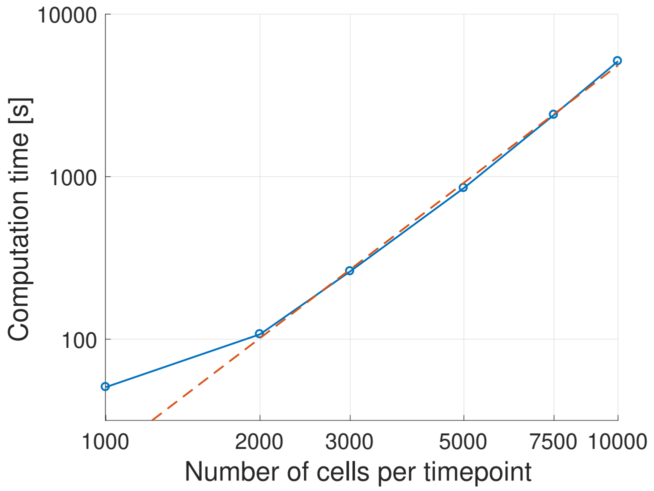

b

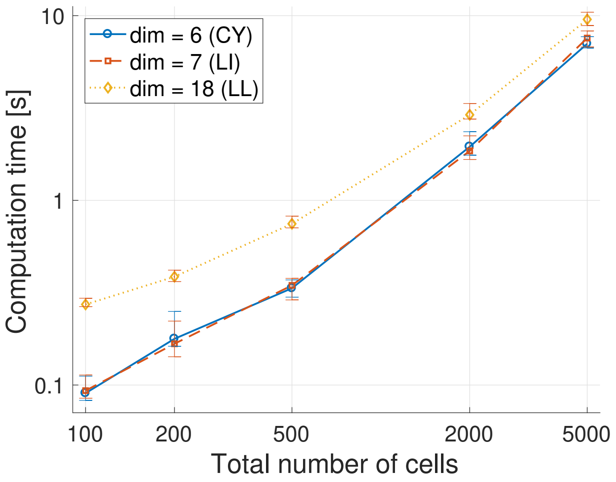

Supplementary figure 13: a: Computation times as a function of number of cells per timepoint in the experiment of Section 3.2 of the main text. The red dashed line is a regression line fitted to the cases with $\geq$ 2000 cells per timepoint. The regression line corresponds to $T \propto N_{\text{cell}}^{2.42}$ where N_{cell} is the number of cells per timepoint. The times were very consistent between the five replicates and therefore only the means are shown in the plot. **b:** Computation times in the BEELINE synthetic dataset for the systems without branching dynamics. The computation time depends on the combination of system dimension and the number of cells. The case where the number of cells is very low (100), should be the most representative on the effect of dimension on computation time. In this case, the increase of the dimension from 6 or 7 to 18 increases the median computation time by a factor of 2.3 ($0.1227s$ or $0.1248s$ to $0.2833s$).
